# Genomic plasticity and adaptive capacity of the quaternary alkyl-ammonium compound and copper tolerant *Acinetobacter bohemicus* strain QAC-21b isolated from pig manure

**DOI:** 10.1007/s10482-022-01805-w

**Published:** 2023-01-16

**Authors:** Dipen Pulami, Lina Schwabe, Jochen Blom, Oliver Schwengers, Gottfried Wilharm, Peter Kämpfer, Stefanie P. Glaeser

**Affiliations:** 1 Institute of Applied Microbiology, Justus-Liebig University Giessen, 35392 Giessen, Germany; 2 Bioinformatics and Systems Biology, Justus-Liebig University Giessen, 35392 Giessen, Germany; 3 Project Group P2, Robert Koch Institute, Wernigerode Branch, 38855 Wernigerode, Germany

**Keywords:** *Acinetobacter bohemicus*, QAAC tolerance, Manure, Environmental transmission

## Abstract

Here, we present the genomic characterization of an *Acinetobacter bohemicus* strain QAC-21b which was isolated in the presence of a quaternary alky-ammonium compound (QAAC) from manure of a conventional German pig farm. The genetic determinants for QAAC, heavy metal and antibiotic resistances are reported based of the whole genome shotgun sequence and physiological growth tests. *A. bohemicus* QAC-21b grew in a species typical manner well at environmental temperatures but not at 37 °C. The strain showed tolerance to QAACs and copper but was susceptible to antibiotics relevant for *Acinetobacter* treatments. The genome of QAC-21b contained several *Acinetobacter* typical QAAC and heavy metal transporting efflux pumps coding genes, but no key genes for acquired antimicrobial resistances. The high genomic content of transferable genetic elements indicates that this bacterium can be involved in the transmission of antimicrobial resistances, if it is released with manure as organic fertilizer on agricultural fields. The genetic content of the strain was compared to that of two other *A. bohemicus* strains*,* the type strain ANC 3994^T^, isolated from forest soil, and KCTC 42081, originally described as *A. pakistanensis,* a metal resistant strain isolated from a wastewater treatment pond. In contrast to the forest soil strain, both strains from anthropogenically impacted sources showed genetic features indicating their evolutionary adaptation to the anthropogenically impacted environments. Strain QAC-21b will be used as model strain to study the transmission of antimicrobial resistance to environmentally adapted *Acinetobacter* in agricultural environments receiving high content of pollutants with organic fertilizers from livestock husbandry.

**Supplementary Information:**

The online version contains supplementary material available at 10.1007/s10482-022-01805-w.

## Introduction

Members of the genus *Acinetobacter* are widespread in nature and have been cultured from both clinical and non-clinical environments including soil, water, wild birds and domestic/farm animals (Towner 2009; Visca et al. 2011; Wilharm et al. 2017; Pulami et al. 2021). The ecology of most of the species is still not well understood (Cool et al. 2019). Multi-drug resistant (MDR) pathogens belonging to the *Acinetobacter calcoaceticus-Acinetobacter baumannii* (ACB) complex have immense clinical significance for causing hospital-associated nosocomial outbreaks globally (Wong et al. 2017; Vázquez-López et al. 2020). The overuse of antibiotics in the clinical environment and livestock husbandry has accelerated the dissemination of antibiotics, antibiotic resistant bacteria (ARB) and antibiotic resistance genes (ARGs) from human populations and livestock into the receiving environment (Chee-Sanford et al. 2009; Rahube et al. 2012; Rizzo et al. 2013; Bürgmann et al. 2018; Xie and Zhao 2018). Beside antibiotic residues, heavy metals and biocides, including quaternary alkyl-ammonium compounds (QAACs), are present in wastewater and manure from livestock husbandry in parallel (Heuer et al. 2011; Rahube et al. 2012; Mulder et al. 2018; Kinney et al. 2006; Christou et al. 2017; Pan and Chu 2018). Those pollutants can trigger the spread of ARGs via co-selection processes in manure and WWTPs or in the environment where bacteria and pollutants come in close contact (Chapman 2003; Imran et al. 2019). Resistance and tolerance to QAACs and heavy metal ions is often associated with mobile genetic elements (MGEs) that have been responsible for a wide dissemination of ARGs (Baker-Austin et al. 2006; Hobman and Crossman 2015; Li et al. 2017). *Acinetobacter* spp. including *A. baumannii* strains have occasionally been isolated from raw manure and digested manure of biogas plant digestates (Schauss et al. 2015, 2016; Pulami et al. 2020). So far, less is known about QAAC resistance of *Acinetobacter* which are released with manure in the agricultural environment. Less is known about *Acinetobacter* from those sources which do not grow at pathogen relevant temperatures because cultivation studies for risk assessment are often performed at higher temperatures as 37 °C or even 44 °C (Schauss et al. 2015, 2016; Klotz et al. 2019; Pulami et al. 2020). We attempted to study the presence of QAAC tolerant bacteria which grew at environmental relevant temperatures in pig manure (data unpublished). The studied manure slurry contained QAACs and was used as fertilizer for agricultural fields. During this study we cultured an *Acinetobacter* strain, QAC-21b, in the presence of 50 µg benzyldimethyldodecylammonium chloride (BAC-C12) mL^−1^. The strain was identified as a member of the species *Acinetobacter bohemicus.* The species was originally proposed based on 25 isolates which were isolated from different soil and water samples collected in natural ecosystems in the Czech Republic (Krizova et al. 2014). In the same year, a second species, *A. pakistanensis* was proposed based on one strain which was isolated from a textile dying wastewater treatment pond in Pakistan (Abbas et al. 2014). This strain was characterized as heavy metal resistant and psychrotolerant. *A. pakistanensis* was later reclassified as heterotypic synonym of *A. bohemicus* (Nemec and Radolfova-Krizova 2016)*.* Based on phylogenomic analysis, *A. bohemicus* belongs together with the next related species “*A. kyonggiensis*”*, A. albensis, A. harbinensis, A. terrestris*, *A. terrae* and *A. kookii* to a clade of *Acinetobacter* species which contains species that have nearly exclusively recovered from soil and water ecosystems (Clade G; Nemec 2022). As reported for most of the species of this clade, *A. bohemicus* strains did not grow at 37 °C (Nemec 2022). Strains of *A. bohemicus* just grew at 30 °C, but not at 35 °C in brain heart infusion broth (Krizova et al. 2014; Nemec and Radolfova-Krizova 2016).

Here we present a detailed physiological and genomic characterization of the BAC-C12 tolerant *A. bohemicus* strain QAC-21b isolated from the manure sample of a German pig farm. Genetic features explaining QAAC and heavy metal tolerance were studied. The presence of further antimicrobial resistance genes, insertion sequence (IS) elements, phages and pathogenicity genes were examined. Physiological tests were performed in parallel to confirm the genetic predictions of antimicrobial resistances. Aim of the detailed characterization was to understand the adaptation of the strain to the anthropogenic environment and to determine the potential risk associated with QAAC tolerant *Acinetobacter* if it is released on an agricultural field. Genome based analyses were performed in comparison to *A. bohemicus* ANC 3994^T^, which was originally isolated from an environmental habitat which had no contact to anthropogenic pollutants and the copper tolerant strain KCTC 42081 (originally described as the type strain of *A. pakistanensis*) also isolated from an anthropogenically impacted environment.

## Material and methods

### Sampling, isolation and initial phylogenetic identification

The studied strain was isolated from a pooled manure sample collected in March 2017 from a manure storage tank on a pig farm in Hesse, Germany. The pigsty on the farm was cleaned with a mixture of water and didecyldimethylammonium chloride, and the mixture drained in the same tank as the manure. Three samples (each 40 mL) were taken in 50 mL sterile screw cup tubes (Greiner Bio-One GmbH, Germany) and transported cooled to 6 °C to the laboratory. Cultivation was performed during the same day. Bacteria were detached from 10 g liquid manure (pooled from the three replicates) by shaking the sample for 5 min at 25 °C in 90 mL 0.2% tetrasodiumpyrophosphate buffer (TSPP; 0.22 µm; filter-sterilized) in a sterile 250 mL glass bottle at 150 rpm on a horizontal shaker. Thereafter, the bottle was stored for 30 min in the dark to enable the sedimentation of manure particles. After sedimentation, cultivation was performed with the bacterial suspension (upper supernatant 30 mL: 10^–1^ dilution). A subsample of 0.5 mL was serially diluted (up to 10^–3^) in 0.9% (w/v) NaCl solution. From each dilution 100 µL were plated on different agar media which were incubated for 72 h in the dark at 25 °C. A round, beige colony with a diameter of 1.5 mm was picked from Mueller–Hinton agar (MH, Carl-Roth, Germany) supplemented with 50 µg mL^−1^ BAC-C12. The colony was purified by streaking single colonies for multiple times and assigned as strain QAC-21b. Fresh biomass of the strain was preserved for long term storage in two 1.4 mL U-bottom push cap tubes (Micronic, Netherlands) with 500 µL Gibco new-born calf serum (NBCS, ThermoFisher Scientific) at −20 and −80 °C. The strain was assigned by partial 16S rRNA gene sequencing as described by Schauss et al. (2015) to the genus *Acinetobacter*. The Sanger sequenced 16S rRNA gene of strain QAC-21b was deposited in GenBank (NCBI) with accession number OM327586.

### Physiological tests for taxonomic characterization

Phenotypic characterization of strain QAC-21b was performed with the API 20 NE system (bioMérieux). The cytochrome-c oxidase activity was tested with the Bactident oxidase test strips (Merck). Hemolysis activity was tested on Columbia agar with 5% sheep blood (SBA; Oxoid) as described by Krizova et al. (2014). All incubations were performed at 25 °C. Temperature dependent growth was tested with the spot assay technique as described by Pulami et al. (2021). Growth in brain heart infusion (BHI) broth (Sigma Aldrich) at 30 °C and 35 °C were performed according to Krizova et al. (2014) and Nemec and Radolfova-Krizova (2016).

### Susceptibility tests against QAACs, copper and antibiotics

The two QAACs, BAC-C12 and didecyldimethylammonium chloride (DADMAC-C10), were used for susceptibility testing of the strain against common biocides present in disinfection solutions used on farms. Minimal inhibitory concentrations (MIC) values were determined by broth microdilution assay following the CLSI guidelines (M100-ED30) as described by Heyde et al. (2020). Following concentration ranges were tested, 0, 3.125, 6.25, 12.5, 25, 50, 100 and 200 µg BAC-C12 mL^−1^, and 0, 0.3125, 0.625, 1.25, 2.5, 5, 10 and 20 µg DADMAC-C10 mL^−1^. Strain QAC-21b was cultured on MH agar over night at 25 °C and suspended in 0.9% sterile NaCl to a turbidity equal to a 0.5 McFarland standard. 61 µL of the suspension was used for the inoculation of 14 mL MH broth. The broth microdilution assay was performed in a 96 wells plate (Greiner Bio-one) in a total volume of 150 µL per test well containing 25 µL double concentrated MH broth, 25 µL water dissolved QAACs (six-fold concentration) and 100 µL of the bacterial suspension in MH broth. The plate was sealed with sterile transparent plastic cover and incubated for 48 h at 25 °C under humid conditions. The lowest concentration that inhibited growth was considered as MIC value of the QAAC. Copper resistance was tested according to Pulami et al. (2020). Susceptibility test against antibiotics was performed in the MRGN Micronaut-S System panel (Merlin, Germany) and a Micronaut S panel (Merlin) containing mainly veterinary relevant antibiotics (Schauss et al. 2015, 2016) following the CLSI guidelines as described previously (Pulami et al. 2020). Classification into sensitive (S) or resistant (R) for antibiotics was done according to EUCAST (http://www.eucast.org/clinical breakpoints/) and CLSI (M100-ED30).

### Genome sequencing, phylogenetic assignment, and genome-wide analyses

Genomic DNA was extracted with the MasterPure DNA purification kit (Epicentre, Madison, Wisconsin, USA) from fresh biomass cultured on MH agar at 25 °C. A DNA library was generated using the Nextera XT DNA sample preparation kit following the manufacturer's instructions. The whole genome shotgun library was sequenced using the dual index paired-end (v3, 2 × 300 bp) approach for the Illumina MiSeq platform as recommended by the manufacturer (Illumina, San Diego, USA). The genome assembly was performed as described previously (Pulami et al. 2021). The genome sequence was deposited in NCBI under accession number CAJJDZ000000000.

Initial phylogenetic analysis based on the partial 16S rRNA gene sequence was performed according to Pulami et al. (2021). A 16S rRNA gene at contig NZ_CAJJDZ010000011 (locus tag: QAC21B_03874) was identical to the Sanger sequence of the 16S rRNA gene and confirmed the strain assignment. For a higher resolution phylogenetic analysis, genes of the DNA-directed RNA polymerase β-subunit (*rpoB*) and the DNA gyrase β-subunit (*gyrB*) were retrieved from genome sequences. Analysis is described in detail in the Supplementary Material.

Comparative genomics including average nucleotide identity (ANI) analyses were performed in EDGAR 3.0 (Blom et al. 2016; Dieckmann et al. 2021). For comparative genomics, genome sequences of the type strains of *A. bohemicus*, *A. pakistanensis* (later heterotypic synonym of *A. bohemicus*; Nemec and Radolfova-Krizova 2016), *A. johnsonii* and *A. kookii*, and *A. baumannii* as well as some well characterized *A. baumannii* strains, AYE, ATCC 17987, and KPC-SM-125 were used. The latter one was isolated from digested manure of German biogas plant (Pulami et al. 2020).

Metal tolerance genes (MTGs) and antibiotic resistance genes were searched using BacMet database (Pal et al. 2014) and Resfinder 4.0 (Zankari et al. 2012; Bortolaia et al. 2020). IslandViewer 4 (Bertelli et al. 2017) for the GI-like regions, and ISfinder (Siguier et al. 2006) was applied to search IS elements. Phage-related sequences were searched using PHASTER (Arndt et al. 2016; Zhou et al. 2011) and plasmids via Plasmidfinder 2.1 (Carattoli et al. 2014). Virulence factor database (VFDB) was used to identify virulence genes (Chen et al. 2005; Liu et al. 2022). Easyfig v2.2.5 (Sullivan et al. 2011) was used to visualize the comparisons of efflux pump operons detected in the genome.

## Results and discussion

### Phylogenetic assignment of strain QAC-21b to the species *A. bohemicus*

The initial phylogenetic identification of QAC-21b showed that the strain shared highest 16S rRNA gene sequence similarity with the type strain of *A. bohemicus* (99.72%) and was placed into the distinct cluster of all *A. bohemicus* strains in a phylogenetic trees based on the 16S rRNA gene (Figure S1, Supplementary data). This was confirmed by the phylogenetic analyses based on the common *Acinetobacter* marker genes *rpoB* and *gyrB* (Figures S2–S4, Supplementary data).

Phylogenomic analysis finally confirmed the species assignment of QAC-21b. ANI values between the genome sequence of QAC-21b and those of *A. bohemicus* ANC 3994^T^ and KCTC 42081 were 98.0 and 98.9%, respectively (Figure S5a). The values were above the 95% threshold for species discrimination (Richter and Rosselló-Móra 2009), and within the range of intraspecies ANI values described for *A. bohemicus* (95.92–96.08%) (Nemec and Radolfova-Krizova 2016). The ANI values indicated that QAC-21b represents a novel strain of the species *A. bohemicus*.

### Comparative genome data

The genome size (3.99 Mbp) and the GC content (39.4%) of the whole genome sequence (WGS) of strain QAC-21b were in a similar range to that of ANC 3994^T^ (3.66 Mbp and 39.6%) and KCTC 42081 (3.7 Mbp and 39.2%) (Table 1). The genome of QAC-21b contained 3943 predicted genes and 3848 coding DNA sequence (CDS). Additional genomic features are provided in Table 1. Strain QAC-21b shared 2639 of 3535 genes (74.5% of the genomic gene content) with the other two strains of the species *A. bohemicus.* Strain QAC-21b shared 77 gene (2.2%) only with ANC 3994^T^ and a higher proportion of gene, 307 genes (8.7%), only with KCTC 42081 (Figure S5b). A total of 519 genes (14.7%) were only detected in the genome of strain QAC-21b. The percentage of genes specific for individual strains was similarity for the two other studied strains.

**Table 1:** Genome assembly and annotation statistics of *A. bohemicus* strains QAC-21b, ANC 3994^T^ and KCTC 42081

Genome features	QAC-21b	ANC 3994^T^	KCTC 42081
GenBank accession number	NZ_CAJJDZ000000000	NZ_APOH00000000	NZ_FOZU00000000
Approximate genome size	3,996,158 bp	3,647,901 bp	3,728,266 bp
BioProject number	PRJEB42457	PRJNA224116	PRJNA224116
BioSample number	SAMEA7810071	SAMN01828192	SAMN05444586
GC content	39.4%	39.6%	39.2%
Genome coverage	22×	87.0×	413×
Number of contigs	17	28	105
Contig N50	902,613 bp	881,473 bp	85,922 bp
Contig L50	2	2	12
Number of scaffolds	17	14	104
Longest contig (bp)	1,907,865 bp	794,304 bp	301,207 bp
Number of predicted genes	3943	3430	3638
Number of predicted CDS	3848	3320	3559
Number of complete rRNAs	11	24	3
Number of partial rRNAs	1	Absent	Absent
Number of predicted tRNAs	78	82	72
Number of non-coding RNAs	5	4	4
Number of pseudogenes	203	72	188
Source	Animal (pigs)	Natural environment	Textile dying wastewater treatment pond
Sample	Pig manure	Deciduous forest soil	Wastewater pond
Country	Germany	Czech Republic	Pakistan
Publication	This study	Krizova et al. (2014)	Abbas et al. (2014)

### Physiological properties of strain QAC-21b confirming species assignment

Physiological properties of strain QAC-21b confirmed the species assignment. Strain QAC-21b was cytochrome-c oxidase negative and did not produce acid from D-glucose, was negative for gelatine hydrolysis and nitrate reduction (API 20 NE system) (Krizova et al. 2014; Nemec and Radolfova-Krizova 2016). Strain QAC-21b was positive for esculin hydrolysis (API 20 NE system) and did not assimilate D-glucose, L-arabinose, malate, phenylacetic acid, D-mannose, D-mannitol, N-acetyl-D-glucosamine, D-maltose, potassium gluconate, capric acid, adipic acid and trisodium citrate. A slight hemolysis was obtained on sheep blood agar. Good growth was observed at 30 °C but not at 35 °C in BHI broth. Spot assays showed an optimum temperature range for growth of QAC-21b between 25 to 30 °C, growth was strongly reduced at 15 °C and no growth was observed at 37 °C (Figure S6). The results were confirm to those reported by Krizova et al. (2014) for *A. bohemicus* strains, most of which showed a weak hemolysis of sheep blood and did not grow at 35 °C.

### Phenotypic resistance of strain QAC-21b to QAAC, copper and common antibiotics

MIC values of QAC-21b for BAC-C12 and DADMAC-C10 were 50 and 2.5 µg mL^−1^. The strain grew on MH agar supplemented with 4 mM copper, but not in the presence of 8 mM copper and above (Figure S7). Strain QAC-21b showed intermediate resistance to tetracycline. However, it was susceptible against amikacin, cefotaxime, ceftazidime, ciprofloxacin, levofloxacin, colistin, imipenem, meropenem, piperacillin, piperacillin/tazobactam and trimethoprim/sulfamethoxazole (Table S1). It showed high MIC values for fosfomycin, oxacillin, ceftiofur (third generation chepalosporine), tylosin and florfenicol (Table S1).

### QAACs and heavy metal efflux, and antimicrobial resistance genes

The genetic determinant often mentioned in context of QAAC tolerance is the gene *qacE* or its mutated version *qacE∆1* (Gaze et al. 2005, 2011). These genes are often co-located with a class 1 integron on plasmids and correlated with reduced susceptibility to QAACs (Fournier et al. 2006; Gomaa et al. 2017; Lin et al. 2017). Both genes were found in clinically relevant *Acinetobacter*, mostly *A. baumannii*. Comparative genomics showed that neither strain QAC-21b nor the next related tested *Acinetobacter* strains contained those genes; *qacE∆1*, co-located with a class 1 integron, was only detected in the *A. baumannii* MDR strain AYE which was isolated from an infected patient (Poirel et al. 2003) (Table S2, Figure S8). The genetic arrangement found in that strain was typical for that gene.

**Fig. 1:**
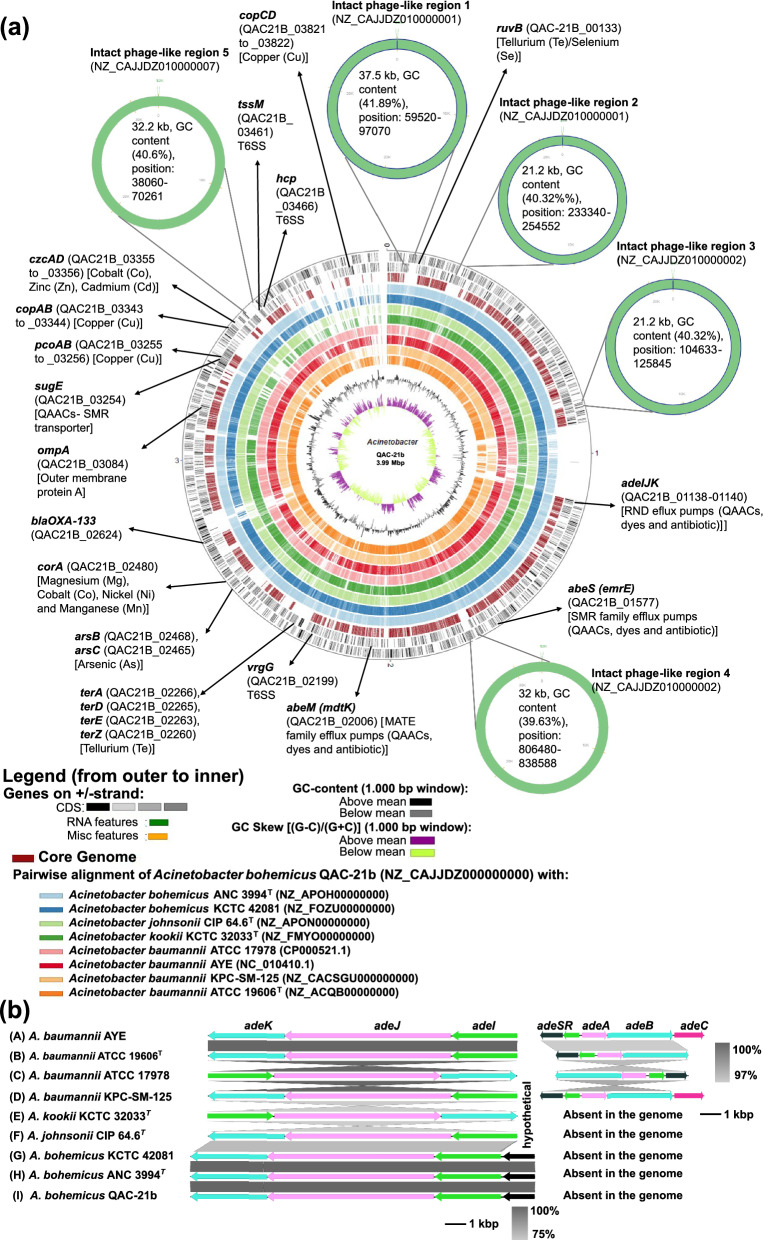
**a** Circular plot of the whole genomes of *A. bohemicus* QAC-21b (size at the centre of plot), *A. bohemicus* ANC 3994^T^, *A. bohemicus* KCTC 42081 (formerly type strain of *A. pakistanensis*), *A. kookii* 32033^T^, *A. johnsonii* CIP 64.6^T^, *A. baumannii* KPC-SM-125, *A. baumannii* ATCC 17,978*, A. baumannii* AYE, and *A. baumannii* ATCC 19606^T^ generated with BioCircos (Cui et al. 2016) implemented in EDGAR3 (Dieckmann et al. 2021). All labelled genes were found to be located at the chromosomes. **b** Comparision of operon of RND efflux pumps (AdeABC/AdeRS and AdeIJK) present in the chromosome of *Acinetobacter* strains, including *A. bohemicus* QAC-21b. Figure and comparison were visualized using Easyfig v2.2.5 (Sullivan et al. 2011)

Search in the BacMet database and comparative genomics showed several genes encoding multiple QAACs and antimicrobial efflux pumps such as multidrug and toxic compound extrusion (MATE) family efflux pumps (*abeM* or *mdtK*), small multidrug resistance (SMR) family efflux pumps (*sugE* and *abeS*) and resistance-nodulation-cell division (RND) efflux pump (*adeIJK*) in the genome of strain QAC-21b and the other *Acinetobacter* strains used for comparative analyses (Fig. 1A, B; Table S2). The combined action of these efflux pumps might be responsible for reduced susceptibility to the tested QAACs. Previous studies showed the association of *adeIJK* with reduced susceptibility to benzalkonium chloride (QAAC) in clinical *A. baumannii* strains (Damier-Piolle et al. 2008; Rajamohan et al. 2010; Lin et al. 2017). *AbeM* is known to be associated with efflux of QAAC in *Acinetobacter* (Su et al. 2005; Lin et al. 2017). Similarly, *abeS* was known to be associated with reduced susceptibility to benzalkonium chloride in clinically associated *A. baumannii* strain AC0037 (Srinivasan et al. 2009). *SugE* was associated with reduced susceptibility to benzalkonium chloride in *Escherichia coli* (Chung et al. 2002; He et al. 2011). We could not determine genes of the RND type efflux pumps AdeABC and AdeRS in the genome of QAC-21b and next related strains, but in all studied *A. baumannii* strains including strain KPC-SM-125, which was isolated from digested manure.

Analysis in BacMet database revealed metal efflux pump genes such as tellurium/selenium efflux genes (*terZ, terE, terD, terA* and *ruvB*)*,* magnesium/cobalt/nickel/manganese efflux genes (*corA*)*,* arsenic efflux genes (*arsC* and *arsB*)*,* zinc/cobalt/cadmium efflux genes (*czcA* and *czcD*), copper resistance protein efflux genes (*pcoA, pcoB, copA, copB, copC* and *copD*) in the genome of QAC-21b (Fig. 1A; Table S2). These genes were reported among the copper tolerant strains of *A. baumannii* (Williams et al. 2016; Thummeepak et al. 2020). Other heavy metals were not tested so far. Tellurium resistance genes were here only detected in strain QAC-21b and the two other *A. bohemicus* strains, but not in the genomes of the other studied strains (Table S2).

The resistome analysis showed that the genome of QAC-21b harbored one gene coding for an intrinsic class D beta-lactamase (Table S2). The high MIC value against oxacillin may be explained by the expression of intrinsic class D β-lactamase gene present in the genome of *Acinetobacter* species (Cho et al. 2018). Naturally weakly expressed class D β-lactamases (*bla*_OXA-type_) become potent if insertion of an IS element occurs upstream (Vandecraen et al. 2017), however QAC-21b lacked an IS element upstream of this gene. The complete sequence of the intrinsic class D beta-lactamase (blaOXA-133, assigned via NCBI genome submission) of QAC-21b had 96.43% (nucleotide based) and 95.90% (protein based) similarity to the intrinsic beta-lactamase of ANC 3994^T^ (*bla*OXA-296, locus tag: F994_00492). The intrinsic class D beta-lactamase of KCTC 42081 was disrupted by an internal stop codon and not assigned further. Perichon et al. (2014) identified a second intrinsic beta-lactamase gene ADC-ANC 3994 (AmpC-type) in the genome of ANC 3994^T^ (logus tag F994_00202). A homologue to this gene was not present in the genomes of QAC-21b and KCTC 42081.

No specific ARGs were determined in the genome of strain QAC-21b. The higher MIC values for the third-generation cephalosporin ceftiofur, florfenicol, fosfomycin and tylosin, and intermediate resistance to tetracycline (Table S1) could be due to the basal-level expression of *adeIJK* and *abeM* (reviewed by Coyne et al. 2011). The intrinsic resistance of *Acinetobacter* to these veterinary (florfenicol and ceftiofur) and clinical (fosfomycin) antibiotics is known (Coyne et al. 2011), however few studies had been performed to use fosfomycin in combination with other drugs, such as colistin, minocycline and polymyxin against *Acinetobacter* (Zhang et al. 2013; Sirijatuphat and Thamlikitkul 2014).

### Plasmid and phage content

The draft genome sequence of QAC-21b lacked plasmids, but contained several phages integrated into the genome. Five intact, five incomplete, and three questionable phage-like regions were detected in the chromosome using PHASTER (Table S3). Similarly, ANC 3994^T^ showed one intact, five incomplete and two questionable phage-like regions (Table S4). Only four incomplete phage-like regions were found in the genome of KCTC 42081 (Table S5). The intact phage-like regions in QAC-21b were similar to the *Mannheimia* phage vB_MhM_3927AP2 (NC_028766.1) and the *Acinetobacter* phage YMC11/11/R3177 (NC_041866.1). In contrast, the intact phage-like region in ANC 3994^T^ was similar to *Burkholderia* phage KS14. The two intact phage-like regions located in contigs NZ_CAJJDZ010000001 (phage region size: 37.5 kb) and NZ_CAJJDZ010000002 (phage region size: 32 kb) of strain QAC-21b carried genes coding for integrases, transposases, terminases, tRNAs, and phage portal, head, tail, plate, fibre and phage-like proteins, including the *attL* and *attR* recognition sites, and multiple hypothetical genes (Figs. 1A, S10). By contrast, the phage region detected in the genome of ANC 3994^T^ lacked genes coding for integrase, transposase and terminase, and the *attL* and *attR* recognition sites were absent (Figure S10). Those genes are required for termination, integration, propagation and lysis inside the host bacterium (Casjens 2003; Canchaya et al. 2003; Labrie et al. 2010). The presence of several phage related genes integrated into the genome of strain QAC-21b was in agreement with the previous findings of multiple phage-linked DNA regions in the genome of *Acinetobacter* species (Touchon et al. 2014). The presence of multiple genes encoding hypothetical proteins in all phage-like regions of strain QAC-21b might be associated with environmental (pig manure) adaptation. These phage-like regions help bacteria to gain antimicrobial resistance, adaptation across changing environments, and can provide novel virulence characteristics to the host bacterium (Brüssow et al. 2004). Multiple studies had reported presence of phages in genome of members of the genus *Acinetobacter*, for instance, *A. baumannii* was considered as polylysogenic as several strains harbored multiple integrated phages in the genome (Snitkin et al. 2011; Touchon et al. 2014; Badawy et al. 2020; Loh et al. 2020).

### IS elements and genomic islands (GIs)

Analysis in the ISfinder showed that strain QAC-21b possessed putative IS elements with 98%, 97% and 98% amino acid sequence similarities to IS*Aba12*, IS*Aba14* and IS*Alw3* (Table S1). These IS elements are often found in the host *A. baumannii* and *A. lwoffii* (Montaña et al. 2016; Liu et al. 2020). IS*Aba14* was reported in clinically relevant European clone II of *A. baumannii* (Šeputienė et al. 2012). Previous studies had shown that IS*Aba12* was responsible for mobilization of the active miniature inverted-repeat transposon element (MITE, designated MITE_*Aba12*_) in the genome of *A. baumannii* ATCC 17978^T^, and disruption of the histone-like nucleoid structuring (*hns*) gene by IS*Aba12* resulted in multiple phenotypic alterations, including hypermotility (Eijkelkamp et al. 2013; Adams and Brown 2019).

**Fig. 2:**
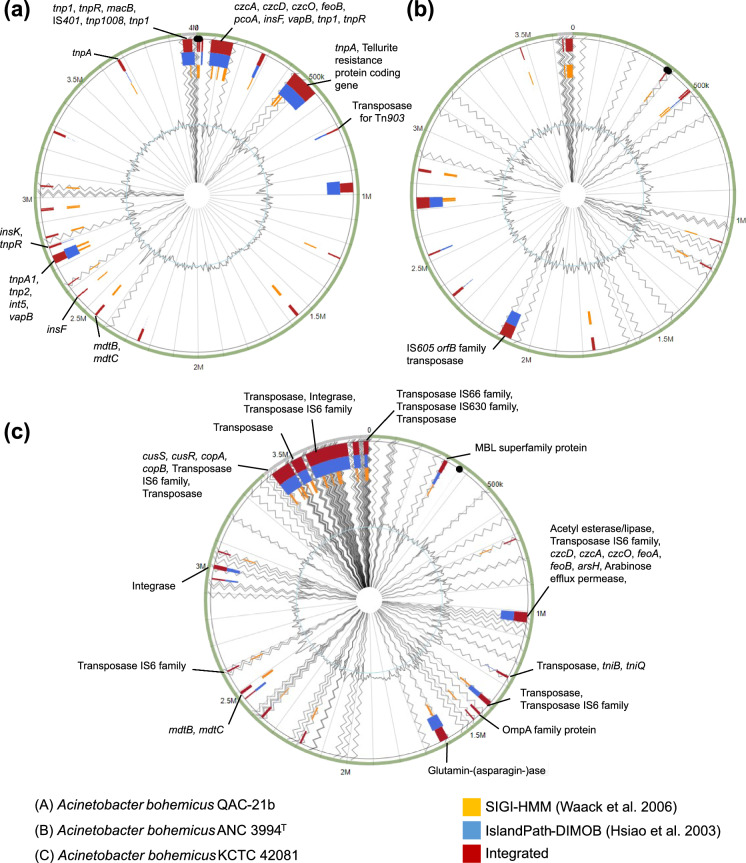
Distribution of putative genomic islands (GIs) in *A. bohemicus* strains QAC-21b (**a**), ANC 3994^T^ (**b**) and KCTC 42081 (**c**). Only, genes associated to metabolism, transposon, IS elements, virulence, metal efflux pumps and antibiotic resistance present within GIs were shown. Genomic islands, which were not labelled, possessed hypothetical and other functional genes. The figures were generated with IslandViewer4. The complete genome of *A. baumannii* ATCC 19606^T^ (NC_CP045110) present in the database of IslandViewer4 was used as reference genome (represented by inner grey circles in the figure). Contig edges were shown by zigzag lines running from the center to the periphery

Based on at least one method of island detection, SIGI-HMM (Waack et al. 2006) or IslandPath-DIMOB (Hsiao et al. 2003), in IslandViewer4, 14 putative genomic islands (GIs) were detected in the genomes of QAC-21b*,* 23 in the genome of ANC 3994^T^ and 21 in the genome of KCTC 42081. GIs of QAC-21b and KCTC 42081 carried genes encoding metal efflux pumps (for e.g., *czcA*, *czcD*, czcO, *pcoA, cusR, cusS, copA, copB, arsH*), antimicrobial resistance (for e.g., *mdtB* and *mdtC*), transposases, integrase, virulence-associated and hypothetical proteins (Table S6) but those of ANC 3994^T^ mostly contained genes coding hypothetical proteins and only a single transposase gene (IS*605* OrfB family; locus tag: F994_02674, TableS6). Microbial GIs are clusters of genes involved in the genome evolution and environmental adaptation. GIs are linked with symbiosis, metabolism, fitness, antimicrobial resistance and pathogenicity (Juhas et al. 2009). The mobile genetic elements (for e.g., transposon, IS elements, plasmids and pathogenicity island) are associated with contemporary rise of antibiotic resistance in *Acinetobacter* species, particularly those representing ACB complex (Peleg et al. 2008; Lean and Yeo 2017). The overview of genes associated with metabolism, transposon, IS elements, virulence, metal efflux pumps and antibiotic resistance present within GIs is provided in Fig. 2 and Table S6. There are several evidences that the spontaneous evolution of ARGs within the genus *Acinetobacter* has been facilitated by IS elements (Turton et al. 2006), integrons (Hujer et al. 2006), conjugative elements (Goldstein et al. 1983) and transformation (Wright et al. 2014).

### Pathogenicity related genes

Strain QAC-21b as well as the next related studied strains possessed a type six secretion system (T6SS). This is a general feature reported from members of genus *Acinetobacter* (Weber et al. 2013, 2016; Lewis et al. 2019). It was reported that *Acinetobacter* often uses T6SS to compete against other bacteria (Weber et al. 2013; Carruthers et al. 2013). The T6SS gene locus in the genome of QAC-21b contained genes encoding the hemolysin coregulated protein Hcp, the valine-glycine repeat protein G (VgrG), the membrane spanning complex (TssM), the baseplate components TssE, TssF and TssG and sheath components TssB and TssC. Apart from T6SS, the search in the virulence factor database showed the presence of other pathogenicity genes encoding outer membrane protein A (OmpA), phospholipase D (PlcD) and two component response regulator transcription factor (BmfRS) (Table S1). Proteins expressed by these genes are involved in adherence, invasion, apoptosis, biofilm, persistence, pathogenesis and serum resistance (Choi et al. 2005, 2008a, 2008b; Gaddy et al. 2009; Lee et al. 2010; Smani et al. 2014; Wang et al. 2014; Jacobs et al. 2010; Tomaras et al. 2008; Liou et al. 2014).

### Survival of anaerobic conditions in the manure storage tank

Several studies showed that the obligate aerobic *Acinetobacter* survived the anaerobic treatment processes in wastewater treatment plants (Higgins et al. 2018) and biogas plants (Schauss et al. 2015, 2016; Pulami et al. 2020, 2021). The slurry tank in the pig farm of this study also represented an anaerobic environment. Comparative genomics showed the presence of genes encoding AMP phosphotransferase (locus tag: QAC21B_01400) and adenylate kinase (QAC21B_01250) in the genome of QAC-21b. Combined enzymatic actions of these proteins help using accumulated phosphates inside the cells as energy source (Van Groenestijn et al. 1987). This explains the survival of obligate aerobic *Acinetobacter* at oxygen limited environments (Pulami et al. 2020, 2021) as it was also expected in the manure slurry tank where the strain was isolates from.

### Knowledge gap on QAAC resistance of *Acinetobacter*

QAACs are common ingredients of pesticides, disinfectants and detergents in agriculture and animal husbandry (Mulder et al. 2018). As reported by the German Society of Veterinary Medicine, at least one quarter of disinfectants used contained QAACs (DVG 2015). Bioavailable concentrations of QAACs can result in cross-resistance (Singer et al. 2016) and co-selection of resistance to antibiotics and QAACs (Webber et al. 2015). However, this important role of QAAC resistance for AMR spread is known less, and little is known about resistance levels developed against QAACs. There is still a gap of knowledge at which point a bacterium must be considered as QAAC susceptible or QAAC resistant to decide if studies on resistance mechanisms are relevant. No respective studied for *Acinetobacter* are published yet. We studied here only one strain and determined MICs for two common QAACs, BAC-C12 and DADMAC-C10. In a recent study we determined the effects of BAC-C12 and DADMAC-C10 exposure of an *A. baumannii* strain KPC-SM-21 isolated from digested manure (Heyde et al. 2020; Pulami et al. 2021). This strain had lower MIC values for the two QAACs (< 5 µg BAC-C12 ml^−1^ and 5 µg DADMAC-C10 ml^−1^) than detected here for strain QAC-21b. This may indicate an increased tolerance of QAC-21b to both QAACs. Previous studies which reported on QAAC resistance mechanisms of *Acinetobacter* strains (Srinivasan et al. 2009; Rajamohan et al. 2010; Lin et al. 2017) did not consider pure QAAC compounds but worked with undefined mixtures of QAACs of different side carbon chain length, which made a direct comparison of our data with those of other studies not possible.

## Conclusion

It has been already indicated that *Acinetobacter* species might contribute to spread and persistence of antimicrobial resistance in manured soil (Leclercq et al. 2016). More detailed studies are still required to understand those processes in detail. Here, we characterized a potential model strain isolated from manure which showed QAAC and copper-tolerance but was still antibiotic susceptible*.* However, the strain showed a high genetic potential to integrate receiving ARGs into its genome. The ability of QAC-21b to grow in a species typical manner well at environmental temperatures but not at 37 °C as typical human pathogenic *Acinetobacter* makes this strain a good model candidate for those environmental studies. Environmentally adapted strains are often neglected in risk assessments of antimicrobial resistance spread, but the presented genomic plasticity of QAC-21b shows that those strains should be considered as vector of transferable resistance determinants which is of concern in the current era of continuous rise in antibiotic resistances influenced by increased anthropogenic activities (Alanis 2005; Ji et al. 2012; Seiler and Berendonk 2012; Furlan et al. 2019). Differences in the structure of GIs and IS elements present in the *A. bohemicus* strains from anthropogenically impacted environments, compared to the type strain of *A. bohemicus* isolated from a native environment indicate adaptation of specific *Acinetobacter* strains of environmental species to human impacted environments. However further proofs including more strains are necessary. Our study indicated that more extensive studies of environmentally adapted *Acinetobacter* strains must be considered to understand the evolutionary adaptation of *Acinetobacter* to anthropogenic contaminations in the environment in much more detail. Strain QAC-21b can serve as a well characterized model strain for those studies.

## Supplementary Information

Below is the link to the electronic supplementary material.

Supplementary file1 (DOCX 12691 kb)

## Data Availability

The Sanger sequenced 16S rRNA gene sequence and the whole genome shotgun sequence of QAC-21b are available at GenBank/EBML/DDBJ under Accession numbers OM327586 and CAJJDZ000000000.

## References

[CR1] Abbas S, Ahmed I, Kudo T (2014). Heavy metal-tolerant and psychrotolerant bacterium *Acinetobacter* pa*kistanensis* sp. nov. isolated from a textile dyeing wastewater treatment pond. Pak J Agric Sci.

[CR2] Adams FG, Brown MH (2019) MITE_*Aba12*_, a Novel mobile miniature inverted-repeat transposable element identified in *Acinetobacter baumannii* ATCC 17978 and its prevalence across the *Moraxellaceae* family. mSphere 4:e00028–19. 10.1128/mspheredirect.00028-19 10.1128/mSphereDirect.00028-19 PMC6382973 30787115

[CR3] Alanis AJ (2005). Resistance to antibiotics: are we in the post-antibiotic era?. Arch Med Res.

[CR4] Arndt D, Grant JR, Marcu A (2016). PHASTER: a better, faster version of the PHAST phage search tool. Nucleic Acids Res.

[CR5] Badawy S, Pajunen MI, Haiko J (2020). Identification and functional analysis of temperate *Siphoviridae* bacteriophages of *Acinetobacter baumannii*. Viruses.

[CR6] Baker-Austin C, Wright MS, Stepanauskas R, McArthur JV (2006). Co-selection of antibiotic and metal resistance. Trends Microbiol.

[CR8] Bertelli C, Laird MR, Williams KP (2017). IslandViewer 4: expanded prediction of genomic islands for larger-scale datasets. Nucleic Acids Res.

[CR9] Blom J, Kreis J, Spänig S (2016). EDGAR 2.0: an enhanced software platform for comparative gene content analyses. Nucleic Acids Res.

[CR10] Bortolaia V, Kaas RS, Ruppe E (2020). ResFinder 4.0 for predictions of phenotypes from genotypes. J Antimicrob Chemother.

[CR11] Brüssow H, Canchaya C, Hardt W-D (2004). Phages and the evolution of bacterial pathogens: from genomic rearrangements to lysogenic conversion. Microbiol Mol Biol Rev.

[CR12] Bürgmann H, Frigon D, Gaze WH, et al (2018) Water and sanitation: an essential battlefront in the war on antimicrobial resistance. FEMS Microbiol Ecol 94:fiy101. 10.1093/femsec/fiy101 10.1093/femsec/fiy101 29878227

[CR13] Canchaya C, Proux C, Fournous G (2003). Prophage genomics. Microbiol Mol Biol Rev.

[CR14] Carattoli A, Zankari E, Garciá-Fernández A (2014). PlasmidFinder and pMLST: in silico detection and typing of plasmid. Antimicrob Agents Chemother.

[CR15] Carruthers MD, Nicholson PA, Tracy EN, Munson RS (2013) *Acinetobacter baumannii* utilizes a type VI secretion system for bacterial competition. PLoS One 8:e59388. 10.1371/journal.pone.0059388 10.1371/journal.pone.0059388 PMC3602014 23527179

[CR16] Casjens S (2003). Prophages and bacterial genomics: what have we learned so far?. Mol Microbiol.

[CR17] Chapman JS (2003) Disinfectant resistance mechanisms, cross-resistance, and co-resistance. In: International biodeterioration and biodegradation, pp 271–276. 10.1016/S0964-8305(03)00044-1

[CR18] Chee-Sanford JC, Mackie RI, Koike S (2009). Fate and transport of antibiotic residues and antibiotic resistance genes following land application of manure waste. J Environ Qual.

[CR19] Chen L, Yang J, Yu J (2005). VFDB: a reference database for bacterial virulence factors. Nucleic Acids Res.

[CR20] Cho GS, Li B, Rostalsky A (2018). Diversity and antibiotic susceptibility of *Acinetobacter* strains from milk powder produced in Germany. Front Microbiol.

[CR21] Choi CH, Hyun SH, Lee JY (2008). *Acinetobacter baumannii* outer membrane protein A targets the nucleus and induces cytotoxicity. Cell Microbiol.

[CR22] Choi CH, Lee EY, Lee YC (2005). Outer membrane protein 38 of *Acinetobacter baumannii* localizes to the mitochondria and induces apoptosis of epithelial cells. Cell Microbiol.

[CR23] Choi CH, Lee JS, Lee YC (2008). *Acinetobacter baumannii* invades epithelial cells and outer membrane protein A mediates interactions with epithelial cells. BMC Microbiol.

[CR24] Christou A, Agüera A, Bayona JM (2017). The potential implications of reclaimed wastewater reuse for irrigation on the agricultural environment: the knowns and unknowns of the fate of antibiotics and antibiotic resistant bacteria and resistance genes: a review. Water Res.

[CR25] Chung YJ, Saier MH (2002). Overexpression of the *Escherichia coli sugE* gene confers resistance to a narrow range of quaternary ammonium compounds. J Bacteriol.

[CR27] Cool P, Nemec A, Kämpfer P, Veneechoutte M (2019) *Acinetobacter*, *Chryseobacterium*, *Moraxella*, and other Nonfermentative Gram-Negative Rods. In: Carroll KC, Pfaller MA, Landry ML, eds, et al. Manual of clinical microbiology. ASM Press. 2 Volume Set, 12th Edition. pp 829–857

[CR28] Coyne S, Courvalin P, Périchon B (2011). Efflux-mediated antibiotic resistance in *Acinetobacter* spp. Antimicrob Agents Chemother.

[CR29] Cui Y, Chen X, Luo H (2016). BioCircos.js: an interactive circos javascript library for biological data visualization on web applications. Bioinformatics.

[CR30] Damier-Piolle L, Magnet S, Brémont S (2008). AdeIJK, a resistance-nodulation-cell division pump effluxing multiple antibiotics in *Acinetobacter baumannii*. Antimicrob Agents Chemother.

[CR31] Dieckmann MA, Beyvers S, Nkouamedjo-Fankep RC (2021). EDGAR3.0: comparative genomics and phylogenomics on a scalable infrastructure. Nucleic Acids Res.

[CR32] DVG (2015) 13. Liste der nach Richtlinien der DVG (4. Auflage sowie 3. Auflage für Übergangszeit) geprüften und als wirksam befundenen Desinfektionsmittel für den Tierhaltebereich (Handelspräparate)

[CR33] Eijkelkamp BA, Stroeher UH, Hassan KA (2013). H-NS Plays a role in expression of *Acinetobacter baumannii* virulence features. Infect Immun.

[CR36] Fournier PE, Vallenet D, Barbe V, et al (2006) Comparative genomics of multidrug resistance in *Acinetobacter baumannii*. PLoS Genet 2:e7. 10.1371/journal.pgen.0020007 10.1371/journal.pgen.0020007 PMC1326220 16415984

[CR37] Furlan JPR, de Almeida OGG, De Martinis ECP, Stehling EG (2019). Characterization of an environmental multidrug-resistant *Acinetobacter seifertii* and comparative genomic analysis reveals co-occurrence of antimicrobial resistance and metal tolerance determinants. Front Microbiol.

[CR38] Gaddy JA, Tomaras AP, Actis LA (2009). The *Acinetobacter baumannii* 19606 OmpA protein plays a role in biofilm formation on abiotic surfaces and in the interaction of this pathogen with eukaryotic cells. Infect Immun.

[CR39] Gaze WH, Abdouslam N, Hawkey PM, Wellington EMH (2005). Incidence of class 1 integrons in a quaternary ammonium compound-polluted environment. Antimicrob Agents Chemother.

[CR40] Gaze WH, Zhang L, Abdouslam NA (2011). Impacts of anthropogenic activity on the ecology of class 1 integrons and integron-associated genes in the environment. ISME J.

[CR41] Goldstein FW, Labigne-Roussel A, Gerbaud G (1983). Transferable plasmid-mediated antibiotic resistance in *Acinetobacter*. Plasmid.

[CR42] Gomaa FAM, Helal ZH, Khan MI (2017). High prevalence of *bla*_NDM-1_, *bla*_VIM_, *qacE*, and *qacEΔ1* genes and their association with decreased susceptibility to antibiotics and common hospital biocides in clinical isolates of *Acinetobacter baumannii*. Microorganisms.

[CR43] He GX, Zhang C, Crow RR (2011). SugE, a new member of the SMR family of transporters, contributes to antimicrobial resistance in *Enterobacter cloacae*. Antimicrob Agents Chemother.

[CR44] Heuer H, Schmitt H, Smalla K (2011). Antibiotic resistance gene spread due to manure application on agricultural fields. Curr Opin Microbiol.

[CR45] Heyde BJ, Glaeser SP, Bisping L (2020). Smectite clay minerals reduce the acute toxicity of quaternary alkylammonium compounds towards potentially pathogenic bacterial taxa present in manure and soil. Sci Rep.

[CR46] Higgins PG, Hrenovic J, Seifert H, Dekic S (2018). Characterization of *Acinetobacter baumannii* from water and sludge line of secondary wastewater treatment plant. Water Res.

[CR48] Hobman JL, Crossman LC (2015). Bacterial antimicrobial metal ion resistance. J Med Microbiol.

[CR51] Hsiao W, Wan I, Jones SJ, Brinkman FSL (2003). IslandPath: aiding detection of genomic islands in prokaryotes. Bioinformatics.

[CR52] Hujer KM, Hujer AM, Hulten EA (2006). Analysis of antibiotic resistance genes in multidrug-resistant *Acinetobacter* sp. isolates from military and civilian patients treated at the Walter Reed Army Medical Center. Antimicrob Agents Chemother.

[CR53] Imran M, Das KR, Naik MM (2019). Co-selection of multi-antibiotic resistance in bacterial pathogens in metal and microplastic contaminated environments: an emerging health threat. Chemosphere.

[CR54] Jacobs AC, Hood I, Boyd KL (2010). Inactivation of phospholipase D diminishes *Acinetobacter baumannii* pathogenesis. Infect Immun.

[CR55] Ji X, Shen Q, Liu F (2012). Antibiotic resistance gene abundances associated with antibiotics and heavy metals in animal manures and agricultural soils adjacent to feedlots in Shanghai, China. J Hazard Mater.

[CR56] Juhas M, Van Der Meer JR, Gaillard M (2009). Genomic islands: tools of bacterial horizontal gene transfer and evolution. FEMS Microbiol Rev.

[CR57] Kinney CA, Furlong ET, Werner SL, Cahill JD (2006). Presence and distribution of wastewater-derived pharmaceuticals in soil irrigated with reclaimed water. Environ Toxicol Chem.

[CR58] Klotz P, Higgins PG, Schaubmar AR (2019). Seasonal occurrence and carbapenem susceptibility of bovine *Acinetobacter baumannii* in Germany. Front Microbiol.

[CR59] Krizova L, Maixnerova M, Sedo O, Nemec A (2014). *Acinetobacter bohemicus* sp. nov. widespread in natural soil and water ecosystems in the Czech Republic. Syst Appl Microbiol.

[CR62] Labrie SJ, Samson JE, Moineau S (2010). Bacteriophage resistance mechanisms. Nat Rev Microbiol.

[CR63] Lean SS, Yeo CC (2017). Small, enigmatic plasmids of the nosocomial pathogen, *Acinetobacter baumannii*: Good, bad, who knows?. Front Microbiol.

[CR64] Leclercq SO, Wang C, Sui Z (2016). A multiplayer game: species of *Clostridium*, *Acinetobacter*, and *Pseudomonas* are responsible for the persistence of antibiotic resistance genes in manure-treated soils. Environ Microbiol.

[CR65] Lee JS, Choi CH, Kim JW, Lee JC (2010). *Acinetobacter baumannii* outer membrane protein a induces dendritic cell death through mitochondrial targeting. J Microbiol.

[CR66] Lewis JM, Deveson Lucas D, Harper M, Boyce JD (2019). Systematic identification and analysis of *Acinetobacter baumannii* type VI secretion system effector and immunity components. Front Microbiol.

[CR67] Li LG, Xia Y, Zhang T (2017). Co-occurrence of antibiotic and metal resistance genes revealed in complete genome collection. ISME J.

[CR68] Lin F, Xu Y, Chang Y (2017). Molecular characterization of reduced susceptibility to biocides in clinical isolates of *Acinetobacter baumannii*. Front Microbiol.

[CR69] Liou ML, Soo PC, Ling SR (2014). The sensor kinase BfmS mediates virulence in *Acinetobacter baumannii*. J Microbiol Immunol Infect.

[CR70] Liu B, Zheng D, Zhou S (2022). VFDB 2022: a general classification scheme for bacterial virulence factors. Nucleic Acids Res.

[CR71] Liu L, Shen P, Zheng B (2020). Comparative genomic analysis of 19 clinical isolates of tigecycline-resistant *Acinetobacter baumannii*. Front Microbiol.

[CR72] Loh B, Chen J, Manohar P (2020). A biological inventory of prophages in *A. baumannii* genomes reveal distinct distributions in classes, length, and genomic positions. Front Microbiol.

[CR73] Montaña S, Schramm STJ, Traglia GM (2016). The genetic analysis of an *Acinetobacter johnsonii* clinical strain evidenced the presence of horizontal genetic transfer. PLoS ONE.

[CR74] Mulder I, Siemens J, Sentek V (2018). Quaternary ammonium compounds in soil: implications for antibiotic resistance development. Rev Environ Sci Biotechnol.

[CR75] Nemec A (2022) Acinetobacter. In: Bergey's manual of systematics of Archaea and Bacteria. Wiley, in association with Bergey's Manual Trust. 10.1002/9781118960608.gbm01203.pub2

[CR79] Nemec A, Radolfova-Krizova L (2016) *Acinetobacter pakistanensis* Abbas et al. 2014 is a later heterotypic synonym of *Acinetobacter bohemicus* Krizova et al. 2014. Int J Syst Evol Microbiol 66:5614–5617. 10.1099/ijsem.0.001530 10.1099/ijsem.0.001530 27692032

[CR80] Pal C, Bengtsson-Palme J, Rensing C (2014). BacMet: antibacterial biocide and metal resistance genes database. Nucleic Acids Res.

[CR81] Pan M, Chu LM (2018). Occurrence of antibiotics and antibiotic resistance genes in soils from wastewater irrigation areas in the Pearl River Delta region, southern China. Sci Total Environ.

[CR82] Peleg AY, Seifert H, Paterson DL (2008). *Acinetobacter baumannii*: emergence of a successful pathogen. Clin Microbiol Rev.

[CR83] Poirel L, Menuteau O, Agoli N (2003). Outbreak of extended-spectrum β-lactamase VEB-1-producing isolates of *Acinetobacter baumannii* in a French hospital. J Clin Microbiol.

[CR84] Pulami D, Schauss T, Eisenberg T, et al (2020) *Acinetobacter baumannii* in manure and anaerobic digestates of German biogas plants. FEMS Microbiol Ecol 96:fiaa176. 10.1093/femsec/fiaa176 10.1093/femsec/fiaa176 32832994

[CR85] Pulami D, Schauss T, Eisenberg T (2021). *Acinetobacter stercoris* sp. nov. isolated from output source of a mesophilic German biogas plant with anaerobic operating conditions. Antonie Van Leeuwenhoek.

[CR86] Périchon B, Goussard S, Walewski V (2014). Identification of 50 class D β-lactamases and 65 Acinetobacter-derived cephalosporinases in *Acinetobacter* spp. Antimicrob Agents Chemother.

[CR87] Rahube TO, Yost CK (2012). Characterization of a mobile and multiple resistance plasmid isolated from swine manure and its detection in soil after manure application. J Appl Microbiol.

[CR88] Rajamohan G, Srinivasan VB, Gebreyes WA (2010). Novel role of *Acinetobacter baumannii* RND efflux transporters in mediating decreased susceptibility to biocides. J Antimicrob Chemother.

[CR89] Richter M, Rosselló-Móra R (2009). Shifting the genomic gold standard for the prokaryotic species definition. Proc Natl Acad Sci USA.

[CR90] Rizzo L, Manaia C, Merlin C (2013). Urban wastewater treatment plants as hotspots for antibiotic resistant bacteria and genes spread into the environment: a review. Sci Total Environ.

[CR93] Schauss T, Glaeser SP, Gütschow A, et al (2015) Improved detection of extended spectrum beta-lactamase (ESBL)-producing *Escherichia coli* in input and output samples of German biogas plants by a selective pre-enrichment procedure. PLoS One 10:e0119791. 10.1371/journal.pone.0119791 10.1371/journal.pone.0119791 PMC4370489 25799434

[CR94] Schauss T, Wings TK, Brunner JS (2016). Bacterial diversity and antibiotic resistances of abundant aerobic culturable bacteria in input and output samples of 15 German biogas plants. J Appl Microbiol.

[CR95] Seiler C, Berendonk TU (2012). Heavy metal driven co-selection of antibiotic resistance in soil and water bodies impacted by agriculture and aquaculture. Front Microbiol.

[CR96] Šeputienė V, Povilonis J, Sužiedeliene E (2012). Novel variants of AbaR resistance islands with a common backbone in Acinetobacter baumannii isolates of European clone II. Antimicrob Agents Chemother.

[CR97] Siguier P, Perochon J, Lestrade L (2006). ISfinder: the reference centre for bacterial insertion sequences. Nucleic Acids Res.

[CR98] Singer AC, Shaw H, Rhodes V, Hart A (2016). Review of antimicrobial resistance in the environment and its relevance to environmental regulators. Front Microbiol.

[CR99] Sirijatuphat R, Thamlikitkul V (2014). Preliminary study of colistin versus colistin plus fosfomycin for treatment of carbapenem-resistant *Acinetobacter baumannii* infections. Antimicrob Agents Chemother.

[CR100] Smani Y, Fab̀rega A, Roca I, et al (2014) Role of OmpA in the multidrug resistance phenotype of *Acinetobacter baumannii*. Antimicrob Agents Chemother 58:1806–1808. 10.1128/AAC.02101-13 10.1128/AAC.02101-13 PMC3957889 24379205

[CR101] Snitkin ES, Zelazny AM, Montero CI (2011). Genome-wide recombination drives diversification of epidemic strains of *Acinetobacter baumannii*. Proc Natl Acad Sci USA.

[CR102] Srinivasan VB, Rajamohan G, Gebreyes WA (2009). Role of AbeS, a novel efflux pump of the SMR family of transporters, in resistance to antimicrobial agents in *Acinetobacter baumannii*. Antimicrob Agents Chemother.

[CR104] Su XZ, Chen J, Mizushima T (2005). AbeM, an H+-coupled *Acinetobacter baumannii* multidrug efflux pump belonging to the MATE family of transporters. Antimicrob Agents Chemother.

[CR105] Sullivan MJ, Petty NK, Beatson SA (2011). Easyfig: a genome comparison visualizer. Bioinformatics.

[CR106] Thummeepak R, Pooalai R, Harrison C (2020). Essential gene clusters involved in copper tolerance identified in *Acinetobacter baumannii* clinical and environmental isolates. Pathogens.

[CR107] Tomaras AP, Flagler MJ, Dorsey CW (2008). Characterization of a two-component regulatory system from *Acinetobacter baumannii* that controls biofilm formation and cellular morphology. Microbiology.

[CR108] Touchon M, Cury J, Yoon EJ (2014). The genomic diversification of the whole *Acinetobacter* genus: Origins, mechanisms, and consequences. Genome Biol Evol.

[CR109] Towner KJ (2009). *Acinetobacter*: an old friend, but a new enemy. J Hosp Infect.

[CR110] Turton JF, Ward ME, Woodford N (2006). The role of ISAba1 in expression of OXA carbapenemase genes in *Acinetobacter baumannii*. FEMS Microbiol Lett.

[CR111] Van Groenestijn JW, Deinema MH, Zehnder AJB (1987). ATP production from polyphosphate in *Acinetobacter* strain 210A. Arch Microbiol.

[CR112] Vandecraen J, Chandler M, Aertsen A, Van Houdt R (2017). The impact of insertion sequences on bacterial genome plasticity and adaptability. Crit Rev Microbiol.

[CR113] Vázquez-López R, Solano-Gálvez SG, Vignon-Whaley JJJ (2020). *Acinetobacter baumannii* resistance: a real challenge for clinicians. Antibiotics.

[CR114] Visca P, Seifert H, Towner KJ (2011). *Acinetobacter* infection - an emerging threat to human health. IUBMB Life.

[CR115] Waack S, Keller O, Asper R (2006). Score-based prediction of genomic islands in prokaryotic genomes using hidden Markov models. BMC Bioinformatics.

[CR117] Wang N, Ozer EA, Mandel MJ, Hauser AR (2014). Genome-wide identification of *Acinetobacter baumannii* genes necessary for persistence in the lung. Mbio.

[CR118] Webber MA, Whitehead RN, Mount M (2015). Parallel evolutionary pathways to antibiotic resistance selected by biocide exposure. J Antimicrob Chemother.

[CR122] Weber BS, Miyata ST, Iwashkiw JA et al (2013) Genomic and functional analysis of the type VI secretion system in *Acinetobacter*. PLoS One 8:e55142. 10.1371/journal.pone.0055142 10.1371/journal.pone.0055142 PMC3554697 23365692

[CR119] Weber BS, Hennon SW, Wright MS (2016). Genetic dissection of the type VI secretion system in *Acinetobacter* and identification of a novel peptidoglycan hydrolase, TagX, required for its biogenesis. Mbio.

[CR130] Wilharm G, Skiebe E, Higgins PG et al (2017) Relatedness of wildlife and livestock avian isolates of the nosocomial pathogen *Acinetobacter baumannii* to lineages spread in hospitals worldwide. Environ Microbiol 19(10):4349–4364. 10.1111/1462-2920.13931 10.1111/1462-2920.13931 28925528

[CR123] Williams CL, Neu HM, Gilbreath JJ (2016). Copper resistance of the emerging pathogen *Acinetobacter baumannii*. Appl Environ Microbiol.

[CR124] Wong D, Nielsen TB, Bonomo RA (2017). Clinical and pathophysiological overview of *Acinetobacter* infections: a century of challenges. Clin Microbiol Rev.

[CR125] Wright MS, Haft DH, Harkins DM (2014). New insights into dissemination and variation of the health care-associated pathogen *Acinetobacter baumannii* from genomic analysis. Mbio.

[CR126] Xie WY, Shen Q, Zhao FJ (2018). Antibiotics and antibiotic resistance from animal manures to soil: a review. Eur J Soil Sci.

[CR127] Zankari E, Hasman H, Cosentino S (2012). Identification of acquired antimicrobial resistance genes. J Antimicrob Chemother.

[CR128] Zhang Y, Chen F, Sun E (2013). In vitro antibacterial activity of combinations of fosfomycin, minocycline and polymyxin B on pan-drug-resistant *Acinetobacter baumannii*. Exp Ther Med.

[CR129] Zhou Y, Liang Y, Lynch KH (2011). PHAST: a fast phage search tool. Nucleic Acids Res.

