# Supplementary material for: Genomic plasticity and adaptive capacity of the quaternary alkyl-ammonium compound and copper tolerant Acinetobacter bohemicus strain QAC-21b isolated from pig manure
Source: Antonie Van Leeuwenhoek. 2023 Jan 16;116(4):327–42. doi: 10.1007/s10482-022-01805-w (PMC10024671; doi:10.1007/s10482-022-01805-w)
Supplement: Supplementary file 1 — Supplementary file1 (DOCX 12691 kb) [file 10482_2022_1805_MOESM1_ESM.docx]

**Supplementary Information to**

**Genomic plasticity and adaptive capacity of the quaternary alkyl-ammonium compound and copper tolerant *Acinetobacter bohemicus* strain QAC-21b isolated from pig manure**

Dipen Pulami^1^, Lina Schwabe^1^, Jochen Blom^2^, Oliver Schwengers^2^, Gottfried Wilharm^3^, Peter Kämpfer^1^, Stefanie P. Glaeser^1*^

**Authors affiliation:**

^1^Institut für Angewandte Mikrobiologie, Justus-Liebig-Universität Giessen, D-35392 Giessen, Germany

^2^Institute for Bioinformatics and Systems Biology, Giessen, D-35392 Giessen, Germany

^3^Project group P2, Robert Koch Institute, Wernigerode Branch, D-38855 Wernigerode, Germany

***Corresponding author**:

Stefanie P. Glaeser, Stefanie.Glaeser@umwelt.uni-giessen.de

**Supplementary Material**

Phylogenetoc analysis base on partial *rpoB* and *gyrB* DNA and amino acid sequences

According to Krizova et al. (2014), a 861 bp variable region corresponding to nucleotide positions 2915–3775 of *rpoB* and a 633/636-bp region corresponding to positions 494–1126 of *gyrB* (numbered according to A. baumannii CIP 70.34T NZ_APRG00000000 gene sequences) were analyzed. Analyses were performed in MEGA7 (Kumar et al. 2016). All described strains of the species *A. bohemicus* and type strains of all next related *Acinetobacter* species were included in the analysis. Sequences were derived from GenBank (NCBI). Nucleotide sequences were aligned according to the correct open-reading frames (ORFs) using ClustalW (Thompson et al. 1994). Phylogenetic trees were calculated with the ML method and the general time reversible model (GTR; Nei and Kumar, 2000) for nucleotide sequences and the JTT matrix-based model (Jones et al. 1992) for amino acid sequences. Pairwise sequence similarities were calculated based on distance values (p-distance method in MEGA7).

**Supplementary Data**

**Detailed phylogenetic characterization of QAC-21b based on partial 16S rRNA gene, *rpoB* and *gyrB* sequences**

This phylogenetic placement of QAC-21b was initially performed based on 16S rRNA gene sequences. A respective phylogenetic tree including all *Acinetobacter* species type strains is shown in Figure S1. This phylogenetic placement was confirmed by *rpoB* and *gyrB* nucleotide (Figure S2A and B) and amino acid (Figure S3 and S4) sequence based analyses. Strain QAC-21b was placed together with *A. bohemicus* ANC 3994^T^, the 24 additional *A. bohemicus* strains published by Krizova et al. (2014) and strain KCTC 42081 (originally proposed as the type strain of *A. pakistanensis*, Abbas et al. 2014) again in a distinct species cluster. Strain QAC-21b shared 98.5–99.5% partial *rpoB* nucleotide sequence similarities with the *A. bohemicus* strains, but only 77.3–90.2% with other *Acinetobacter* species*.* This was in agreement with previously published data (Nemec et al. 2009, 2011; Krizova et al. 2014) which showed that *rpoB* sequences of the same *Acinetobacter* species are above 97% identity, whereas the *rpoB* interspecies similarity values are mostly below 95%. The amino acid sequences of the partial *rpoB* gene (from positions 973 to 1258 of RpoB of *A. baumannii* CIP 70.34^T^) were identical for strain QAC-21b and most *A. bohemicus* strains; only strain ANC 4250 showed a single amino acid difference (26T>S).

The *gyrB* similarity values for QAC-21b and the *A. bohemicus* strains ranged from 91.7–98.4% which was within the intra-species range reported by Krizova et al. (2014). Similarity values with other members of the genus *Acinetobacter* were in the range of 72.9–84.8%. In agreement with Krizova et al. (2014), all 25 *A. bohemicus* strains from Krizova et al. (2014) showed a codon insertion (GTT) between positions 774 and 775 of the *gyrB* sequence of *A. baumannii* CIP 70.34^T^, which resulted in an additional valine residue in the GyrB protein. Strains QAC-21b and KCTC 42081 had a different codon insertion (TAT) at this position resulting in the presence of a tyrosine residue.

**Supplementary Tables**

**Table S1.** Minimum inhibitory concentration (MIC) values of strain QAC-21b against QAACs and antibiotics given in µg mL^-1^. MIC value assigned to S, Susceptible, I, Intermediate, or R, Resistant according to CLSI or EUCAST database. -, no data available.

| **Tested compounds** | ***A. bohemicus* QAC-21b**  **MIC [µg ml^-1^]** | ***A. bohemicus* QAC-21b**  **S, I, R assignment according to CLSI** |
| --- | --- | --- |
| **QAACs** |  |  |
| BAC-C12 | 50 | - |
| DADMAC-C10 | 2.5 | - |
| **Antibiotics** |  |  |
| Amikacin | <0.5 | S |
| Cefotaxime | 2 | S |
| Ceftazidime | 2 | S |
| Ceftazidime/3-APB | 2 | - |
| Ceftazidime/Avibactam | <1/4 | - |
| Ceftolozane/Tazobactam | <1/4 | - |
| Chloramphenicol | <8 | - |
| Ciprofloxacin | <0.25 | S |
| Colistin | <1 | S |
| Fosfomycin | >64 | - |
| Imipenem | <1 | S |
| Levofloxacin | <0.5 | S |
| Meropenem | <0.125 | S |
| Meropenem/3-APB | 0.5 | - |
| Meropenem/EDTA | <0.25 | - |
| Piperacillin | <8 | S |
| Piperacillin/Tazobactam | <4/4 | S |
| Temocillin | <32 | - |
| Tigecycline | <0.25 | - |
| Trimethoprim/Sulfamethoxazole | <1/9 | S |
| Amoxicillin | <0.5 | - |
| Oxacillin | 16 | - |
| Ceftiofur | 8 | - |
| Ceftiofur/clavulanic acid | <0.25/4 | - |
| Cefquinom | 0.5 | - |
| Cefquinom/clavulanic acid | <0.25/4 | - |
| Enrofloxacin | <0.0625 | - |
| Tetracycline | 8 | I |
| Tylosin | >16 | - |
| Florfenicol | 8 | - |
| Sulfamethoxazole | <4 | - |

**Table S2.** Comparative genomics of QAC-21b with next related type strains of *Acinetobacter* species and *A. baumannii* strains including the type strain and environmental and clinical strains

| **Strains** | ***A. bohemicus* QAC-21b** | ***A. bohemicus* ANC 3994^T^** | ***A. bohemicus* KCTC 42081** | ***A. johnsonii* CIP 64.6^T^** | ***A. kookii* KCTC 32033^T^** | ***A. baumannii* ATCC 17978** | ***A. baumannii* AYE** | ***A. baumannii* KPC-SM-125** | ***A. baumannii* ATCC 19606^T^** |
| --- | --- | --- | --- | --- | --- | --- | --- | --- | --- |
| **Isolation source** | Pig manure | Deciduous forest soil (Czech Republic) | Textile dyeing wastewater treatment pond Pakistan:Islamabad, Industrial area, Kohnoor mill bioremediation pond | Human duodenum | Soil | Human cerebrospinal fluid | Patient with urinary tract infection | Biogas plant digestate | Urine |
| **Reference** | This study | Krizova et al. (2014) | Abbas et al. (2014) | Bovet and Grimont (1986) | Choi et al. (2013) | Bovet and Grimont, 1986 | Poirel et al. (2003) | Pulami et al. (2020) | Bovet and Grimont (1986) |
| **NCBI Genome accession number** | NZ_CAJJDZ000000000 | NZ_APOH00000000 | NZ_FOZU00000000 | NZ_APON00000000 | NZ_FMYO00000000 | CP000521.1 | NC_010410.1 | NZ_CACSGU000000000 | NZ_ACQB00000000 |
| **Genetic resistance determinants (Accession number)** |  |  |  |  |  |  |  |  |  |
|  |  |  |  |  |  |  |  |  |  |
|  |  |  |  |  |  |  |  |  |  |
| **Efflux pumps genes (Accession number)** |  |  |  |  |  |  |  |  |  |
| SMR family efflux QacE∆1  (QAACs efflux pump) | Absent | Absent | Absent | Absent | Absent | Absent | *qacE∆1* (CT025832) | Absent | Absent |
| Multidrug and toxic compound extrusion (MATE) family efflux AbeM (QAACs, dyes and antibiotic efflux pumps) | *abeM (mdtK)* (QAC21B_02006) | *abeM* (F994_00296) | *abeM* (SAMN05444586_1006117) | *abeM* (F986_03240) | *abeM* (SAMN05421732_10810) | *abeM* (A1S_0395) | *abeM* (ABAYE3381) | *abeM* (ABKPCSM125_00210) | *abeM* (HMPREF0010_01847) |
| Small multidrug resistance (SMR) family efflux AbeS (QAACs, dyes and antibiotic efflux pumps) | *abeS (emrE)* (QAC21B_01577) | *abeS* (F994_02286) | *abeS* (SAMN05444586_100969) | *abeS* (F986_00511) | *abeS* (SAMN05421732_101897) | *abeS* (A1S_2298) | *abeS* (ABAYE1181) | *abeS* (ABKPCSM125_01695) | *abeS* (HMPREF0010_00032) |
| SMR family efflux SugE (QAACs and dyes efflux pumps) | *sugE* (QAC21B_03254) | *sugE* (F994_00697) | *sugE* (SAMN05444586_101190) | *sugE* (F986_01553) | *sugE* (SAMN05421732_101897) | *sugE* (A1S_0710) | *sugE* (ABAYE3107) | *sugE* (ABKPCSM125_03091) | *sugE* (HMPREF0010_01213) |
| Resistance nodulation division (RND) type efflux AdeIJK (QAACs, dyes and antibiotic efflux pump) | *adeIJK* (*oprM*) [(QAC21B_01138 to,_01140) | *adeIJK* (F994_00903 to _00905) | *adeIJK* (SAMN05444586_100846 to _100848) | *adeIJK* (F986_01786 to _01788) | *adeIJK* (SAMN05421732_10556 to _10558) | *adeIJK* (A1S_2735 to _2737) | *adeIJK* (ABAYE0746 to ABAYE0748) | *adeIJK* (ABKPCSM125_00755 to _00757) | *adeIJK* (HMPREF0010_02880 to _02882) |
| RND type efflux AdeABC and AdeRS (antibiotic efflux pump) | Absent | Absent | Absent | Absent | Absent | *adeAB*(A1S_1750 to 1754), AdeRS () | *adeABC* (ABAYE1821 to ABAYE1823), *adeRS* (ABAYE1819 to ABAYE1820) | *adeABC* (ABKPCSM125_02330 to _02332), *adeRS* (ABKPCSM125_02328 to _02329) | *adeAB* (HMPREF0010_00593 to _00594), *adeRS* (HMPREF0010_00591 to _00592), |
|  |  |  |  |  |  |  |  |  |  |
| Tellurium resistance protein TerZ [Tellurium (Te)] | *terZ* (QAC21B_02260) | *terZ* (F994_02978) | *terZ* (SAMN05444586_10109) | Absent | Absent | Absent | Absent | Absent | Absent |
| Tellurium resistance protein TerE [Tellurium (Te)] | *terE* (QAC21B_02263) | *terE* (F994_02981) | *terE* (SAMN05444586_101012) | Absent | Absent | Absent | Absent | Absent | Absent |
| Tellurium resistance protein TerD [Tellurium (Te)] | *terD* (QAC21B_02265) | *terD* (F994_02983) | *terD* (SAMN05444586_101014) | Absent | Absent | Absent | Absent | Absent | Absent |
| Tellurium resistance protein TerA [Tellurium (Te)] | *terA* (QAC21B_02266) | *terA* (F994_02984) | *terA* (SAMN05444586_101015) | Absent | Absent | Absent | Absent | Absent | Absent |
| ATP-dependent DNA helicase RuvB [Tellurium (Te)/Selenium (Se)] | *ruvB* (QAC21B_00133) | *ruvB* (F994_01894) | *ruvB* (SAMN05444586_101477) | *ruvB* (F986_00963) | *ruvB* (SAMN05421732_101574) | *ruvB* (A1S_2588) | *ruvB* (ABAYE0909) | *ruvB* (ABKPCSM125_01441) | *ruvB* (HMPREF0010_02149) |
| CorA metal ion transporter (MIT) family [Magnesium (Mg), Cobalt (Co), Nickel (Ni) and Manganese (Mn)] | *corA* (QAC21B_02480) | *corA* (F994_00277) | *corA* (SAMN05444586_10137) | *corA* (F986_03218) | *corA* (SAMN05421732_11310) | *corA* (A1S_3098) | *corA* (ABAYE0390) | *corA* (ABKPCSM125_03558) | *corA* (HMPREF0010_02609) |
| Arsenate reductase ArsC [Arsenic (As)] | *arsC* (QAC21B_02465) | *arsC* (F994_00973) | *arsC* (SAMN05444586_102917) | *arsC* (F986_01391) | *arsC* (SAMN05421732_10480) | *arsC* (A1S_1452) | *arsC* (ABAYE2200) | *arsC* (ABKPCSM125_02791) | (HMPREF0010_00924) |
| Arsenite resistance protein ArsB [Arsenic (As)] | *arsB* (QAC21B_02468) | *arsB* (F994_00976) | *arsB* (SAMN05444586_102914) | *arsB* (F986_01388) | *arsB* (SAMN05421732_10475) | *arsB* (A1S_1454) | *arsB* (ABAYE2198) | *arsB* (ABKPCSM125_02793) | (HMPREF0010_00922) |
| Cobalt (Co), Zinc (Zn), Cadmium (Cd) efflux pumps | *czcA* (QAC21B_03355) | *czcA* (F994_01136) | *czcA* (SAMN05444586_104711) | *czcA* (F986_00575) | Absent | *czcA* (A1S_3217) | *czcA* (ABAYE0271) | *czcA* (ABKPCSM125_03438) | *czcA* (HMPREF0010_02496) |
| Cobalt (Co) and Zinc (Zn) efflux pumps | *czcD* (QAC21B_03356) | *czcD* (F994_00374) | *czcD* (SAMN05444586_104712) | *czcD* (F986_00579) | *czcD* (SAMN05421732_10894) | *czcD* (A1S_3214) | *czcD* (ABAYE0272) | *czcD* (ABKPCSM125_03439) | *czcD* (HMPREF0010_02497) |
| Copper resistance protein (PcoAB, CopA, CopB, CopC, CopD) [Copper (Cu)] | *pcoAB* (QAC21B_03255 to _03256), *copC* (QAC21B_03822), *copD* (QAC21B_03821)*, copA* (QAC21B_03343), *copB* (QAC21B_03344) | *pcoAB* (F994_00695 to _00696), *copC* (Absent), *copD* (Absent), *copA* (Absent), *copB* (Absent) | *pcoAB* (SAMN05444586_101191 to _101192), *copC* (SAMN05444586_10522), *copD* (SAMN05444586_10521), *copA* [(SAMN05444586_105211), *copB* (SAMN05444586_105212) | *pcoAB* (Absent), *copA* (F986_01556), *copB* (F986_01975), *copD* (F986_01962), *copC* (F986_01963) | *pcoAB* (SAMN05421732_101898 to _101899), *copD* (Absent), *copC* (Absent), *copA* (Absent), *copB* (Absent) | *pcoAB* (A1S_0707 to _0708), *copC* (A1S_2940), *copD* (A1S_2941), *copB* (A1S_2935), *copA* (A1S_2936) | *pcoAB* (ABAYE3110 to ABAYE3111), *copC* (ABAYE3206), *copD* (ABAYE3207), *copB* (ABAYE3200), *copA* (ABAYE3201) | *pcoAB* (ABKPCSM125_03093 to _03094), *copD* (Absent), *copC* (Absent), *copA* (Absent), *copB* (Absent) | *pcoAB* (HMPREF0010_01210 to _01211), *copA* (Absent), *copB* (Absent), *copC* (Absent), *copD* (Absent) |
| **Further resistance determinants** |  |  |  |  |  |  |  |  |  |
| Class D type intrinsic β-lactamse (intrinsic) | *bla*_OXA-133_ (QAC21B_02624) | *bla*_OXA-296_ (F994_00492) | Frameshifted; internal stop codon (SAMN05444586_104315 and 16)* | *bla*_OXA-281_ (F986_02727) | Absent | *bla*_OXA-95_ (A1S_1517) | *bla*_OXA-69_ (ABAYE2122) | *bla*_OXA-909_ (ABKPCSM125_02591) | *bla*_OXA-98_ (HMPREF0010_00844) |
|  |  |  |  |  |  |  |  |  |  |
| **Insertion sequence (IS) element** |  |  |  |  |  |  |  |  |  |
| **Putative IS element 1** |  |  |  |  |  |  |  |  |  |
| Locus tag number | (QAC21B_03920) | Absent | Absent | (F986_01630) | Absent | Absent | Absent | Absent | Absent |
| DNA similarity | 94% to IS*Aba14* (IS family: IS3, origin: *A. baumannii*) |  |  |  |  |  |  |  |  |
| Amino acids similarity | 97% to IS*Aba14* |  |  |  |  |  |  |  |  |
| Coverage | 54% ORF of IS*Aba14* |  |  |  |  |  |  |  |  |
| **Putative IS element 2** |  |  |  |  |  |  |  |  |  |
| Locus tag number | (QAC21B_03923) | Absent | (SAMN05444586_11041) | (F986_00702) | Absent | (A1S_0628) | Absent | Absent | Absent |
| DNA similarity | 99% to IS*Aba12* (IS family: IS5, origin: *A. baumannii*) |  |  |  |  |  |  |  |  |
| Amino acids similarity | 98% to IS*Aba12* |  |  |  |  |  |  |  |  |
| Coverage | 100% ORF of IS*Aba12* |  |  |  |  |  |  |  |  |
| **Putative IS element 3** |  |  |  |  |  |  |  |  |  |
| Locus tag number | (QAC21B_03925) | (F994_02663) | Absent | (F986_00558) | Absent | Absent | Absent | Absent | Absent |
| DNA similarity | 99% to IS*Alw3* (IS family: IS1, origin: *A. lwoffii*) |  |  |  |  |  |  |  |  |
| Amino acids similarity | 98% to IS*Alw3* |  |  |  |  |  |  |  |  |
| Coverage | 100% ORF of IS*Alw3* |  |  |  |  |  |  |  |  |
| **Type 6 secretion system (T6SS) linked genes and other virulence factors** |  |  |  |  |  |  |  |  |  |
| Hemolysin coregulated protein (Hcp) | *hcp* (QAC21B_03466) | *hcp* (F994_02474) | *hcp* (SAMN05444586_100437) | *hcp* (F986_00533) | *hcp* (SAMN05421732_10124) | *hcp* (A1S_1296) | *hcp* (ABAYE2413) | *hcp* (ABKPCSM125_02612) | *hcp* (HMPREF0010_01123) |
| Valine-glycine repeat protein G (VgrG) | *vgrG* (QAC21B_02199) | *vgrG* (F994_02954) | *vgrG*, Frameshifted; internal stop codon (SAMN05444586_102840) | *vgrG* ( F986_02152) | *vgrG* (SAMN05421732_10733) | *vgrG* (A1S_3364) | *vgrG* (ABAYE0118) | *vgrG* (ABKPCSM125_01237) | *vgrG* (HMPREF0010_03005) |
| Membrane spanning complex protein TssM | *tssM* (QAC21B_03461) | *tssM* (F994_02479) | *tssM* (SAMN05444586_100442) | *tssM* (F986_00538) | *tssM* (SAMN05421732_10119) | *tssM* (A1S_1302) | *tssM* (ABAYE2408) | *tssM* (ABKPCSM125_02617) | *tssM* (HMPREF0010_01118) |
| Baseplate components T6SS protein TssE | *tssE* (QAC21B_03465) | *tssE* (F994_02475) | *tssE* (SAMN05444586_100438) | *tssE* (F986_00534) | *tssE* (SAMN05421732_10123) | *tssE* (A1S_1297) | *tssE* (ABAYE2412) | *tssE* (ABKPCSM125_02613) | *tssE* (HMPREF0010_01122) |
| Baseplate components T6SS protein TssF | *tssF* (QAC21B_03464) | *tssF* (F994_02476) | *tssF* (SAMN05444586_100439) | *tssF* (F986_00535) | *tssF* (SAMN05421732_10122) | *tssF* (A1S_1299) | *tssF* (ABAYE2411) | *tssF* (ABKPCSM125_02614) | *tssF* (HMPREF0010_01121) |
| Baseplate components T6SS protein TssG | *tssG* (QAC21B_03463) | *tssG* (F994_02477) | *tssG* (SAMN05444586_100440) | *tssG* (F986_00536) | *tssG* (SAMN05421732_10121) | *tssG* (A1S_1300) | *tssG* (ABAYE2410) | *tssG* (ABKPCSM125_02615) | *tssG* (HMPREF0010_01120) |
| Sheath components T6SS protein TssB | *tssB* (QAC21B_03468) | *tssB* (F994_02472) | *tssB* (SAMN05444586_100435) | *tssB* (F986_00531) | *tssB* (SAMN05421732_10126) | *tssB* (A1S_1294) | *tssB* (ABAYE2415) | *tssB* (ABKPCSM125_02610) | *tssB* (HMPREF0010_01125) |
| Sheath components T6SS protein TssC | *tssC* (QAC21B_03467) | *tssC* (F994_02473) | *tssC* (SAMN05444586_100436) | *tssC* (F986_00532) | *tssC* (SAMN05421732_10125) | *tssC* (A1S_1295) | *tssC* (ABAYE2414) | *tssC* (ABKPCSM125_02611) | *tssC* (HMPREF0010_01124) |
| Membrane associated protein for reduced permeability (intrinsic OmpA) | *ompA* [(QAC21B_03084) | *ompA* (F994_01549) | *ompA* (SAMN05444586_101243) | *ompA* (F986_01636) | *ompA* (SAMN05421732_11053) | *ompA* (A1S_2840) | *ompA* (ABAYE0640) | *ompA* (ABKPCSM125_00647) | *ompA* (HMPREF0010_02782) |
| **Phospholipase D (PlcD)** | *plcD* (QAC21B_03108) | *plcD* (F994_01528) | *plcD* (SAMN05444586_101221) | *plcD* (F986_01612) | *plcD* (SAMN05421732_11074) | *plcD* (A1S_2989) | *plcD* (ABAYE0498) | *plcD* (ABKPCSM125_03038) | *plcD* (HMPREF0010_03706) |
| **Two component response regulator transcription factor (BmfRS)** | *bmfRS* (QAC21B_02363 to _02364) | *bmfRS* (F994_00745 to _00746) | *bmfRS* (SAMN05444586_101142 to _101143) | *bmfRS* (F986_01756 to _01757) | *bmfRS* (SAMN05421732_101839 to _101840) | *bmfRS* (A1S_0748 to _0749) | *bmfRS* (ABAYE3063 to ABAYE3064) | *bmfRS* (ABKPCSM125_00534 to _00535) | *bmfRS* ( HMPREF0010_01249 to _01250) |

**Table S3.** Potential phage genes of *A. bohemicus* QAC-21b. Contig sequences were examined for phage related genes using PHASTER (https://phaster.ca/; Zhou et al. 2011; Arndt et al. 2016).

| **No.** | **Contig Accession** | **Region length** | **Completeness** | **Score** | **Position** | **Most common phage** | **Phage accession** | **GC (%) content** |
| --- | --- | --- | --- | --- | --- | --- | --- | --- |
| 1 | NZ_CAJJDZ010000001 | 37.5 kb | **Intact** | 150 | 59520-97070 | PHAGE_Mannhe_vB_MhM_3927AP2 | NC_028766 | 41.89% |
| 2 | NZ_CAJJDZ010000001 | 21.2 kb | **Intact** | 140 | 233340-254552 | PHAGE_Burkho_KS14 | NC_015273 | 40.32% |
| 3 | NZ_CAJJDZ010000001 | 5.6 kb | Incomplete | 30 | 622985-628631 | PHAGE_Pseudo_H66 | NC_042342 | 36.28% |
| 4 | NZ_CAJJDZ010000002 | 21.2 kb | **Intact** | 140 | 104633-125845 | PHAGE_Burkho_KS14 | NC_015273 | 40.32% |
| 5 | NZ_CAJJDZ010000002 | 32 kb | **Intact** | 140 | 806480-838588 | PHAGE_Acinet_YMC11/11/R3177 | NC_041866 | 39.63% |
| 6 | NZ_CAJJDZ010000003 | 31.6 kb | Incomplete | 60 | 140679-172329 | PHAGE_Acinet_vB_AbaS_TRS1 | NC_031098 | 38.19% |
| 7 | NZ_CAJJDZ010000004 | 7.1 kb | Incomplete | 50 | 13960-21133 | PHAGE_Salini_M8CC_19 | NC_042349 | 39.95% |
| 8 | NZ_CAJJDZ010000007 | 32.2 kb | **Intact** | 110 | 38060-70261 | PHAGE_Acinet_YMC11/11/R3177 | NC_041866 | 40.60% |
| 9 | NZ_CAJJDZ010000010 | 18.1 kb | Questionable | 80 | 22213-40401 | PHAGE_Acinet_Bphi_B1251 | NC_019541 | 40.98% |
| 10 | NZ_CAJJDZ010000010 | 40.2 kb | Questionable | 80 | 74140-114354 | PHAGE_Stenot_S1 | NC_011589 | 37.47% |
| 11 | NZ_CAJJDZ010000010 | 18 kb | Questionable | 70 | 116691-134719 | PHAGE_Stx2_II | NC_004914 | 38.05% |
| 12 | NZ_CAJJDZ010000010 | 20.2 kb | Incomplete | 60 | 139167-159455 | PHAGE_Stx2_c_1717 | NC_011357 | 39.03% |
| 13 | NZ_CAJJDZ010000014 | 11.3 kb | Incomplete | 60 | 3251-14616 | PHAGE_Escher_RCS47 | NC_042128 | 39.16% |

**Table S4**  Potential phage genes of *A. bohemicus* ANC 3994^T^. Contig sequences were examined for phage related genes using PHASTER.

| **No.** | **Contig Accession** | **Region length** | **Completeness** | **Score** | **Position** | **Most common phage** | **Phage accession** | **GC (%) content** |
| --- | --- | --- | --- | --- | --- | --- | --- | --- |
| 1 | NZ_APOH01000003 | 6.9 kb | Incomplete | 10 | 22190-29111 | PHAGE_Escher_phAPEC8 | NC_020079 | 35.09% |
| 2 | NZ_APOH01000003 | 9.5 kb | Incomplete | 30 | 89712-99284 | PHAGE_Gordon_Schwabeltier | NC_031255 | 43.16% |
| 3 | NZ_APOH01000010 | 8.2 kb | Incomplete | 10 | 73071-81279 | PHAGE_Tenaci_pT24 | NC_049383 | 41.92% |
| 4 | NZ_APOH01000015 | 21.2 kb | **Intact** | 130 | 205705-226951 | PHAGE_Burkho_KS14 | NC_015273 | 39.75% |
| 5 | NZ_APOH01000017 | 22.7 kb | Questionable | 70 | 32423-55137 | PHAGE_Escher_RCS47 | NC_042128 | 36.61% |
| 6 | NZ_APOH01000021 | 30.6 kb | Questionable | 80 | 19277-49889 | PHAGE_Pseudo_F116 | NC_006552 | 39.93% |
| 7 | NZ_APOH01000023 | 11.7 kb | Incomplete | 50 | 20537-32297 | PHAGE_Acinet_vB_AbaM_ME3 | NC_041884 | 38.09% |
| 8 | NZ_APOH01000025 | 9.4 kb | Incomplete | 10 | 8572-18026 | PHAGE_Bacill_SP_15 | NC_031245 | 39.79% |

**Table S5.** Potential phage genes of *A. bohemicus* KCTC 42081. Contig sequences were examined for phage related genes using PHASTER.

| **No.** | **Contig Accession** | **Region length** | **Completeness** | **Score** | **Position** | **Most common phage** | **Phage accession** | **GC (%) content** |
| --- | --- | --- | --- | --- | --- | --- | --- | --- |
| 1 | NZ_FOZU01000016 | 24.9 Kb | Incomplete | 40 | 7425-32334 | PHAGE_Shigel_Sf6 | NC_005344 | 37.53% |
| 2 | NZ_FOZU01000020 | 5.3Kb | Incomplete | 10 | 8977-14341 | PHAGE_Acinet_Bphi_B1251 | NC_019541 | 36.05% |
| 3 | NZ_FOZU01000029 | 21.7Kb | Incomplete | 30 | 9074-30841 | PHAGE_Escher_RCS47 | NC_042128 | 40.57% |
| 4 | NZ_FOZU01000055 | 8.6Kb | Incomplete | 40 | 1762-10404 | PHAGE_Acinet_vB_AbaS_TRS1 | NC_031098 | 40.53% |

**Table S6.** List of genes associated to metabolism, transposon, IS elements, virulence, metal efflux pumps and antibiotic resistance present within putative genomic islands (GIs) of strain QAC-21b, [exception: genes encoding hypothetical proteins and other functional genes present in the putative GIs were not listed]. The GIs were predicted using IslandViewer4 (Bertelli et al. 2017).

|  |  |  |  | Putative GIs region in the genome predicted by IslandViewer4 | |
| --- | --- | --- | --- | --- | --- |
| Strain | Gene | Product | Locus tag | Start | End |
| *Acinetobacter bohemicus* strain QAC-21b | *czcA* | Cobalt-zinc-cadmium resistance protein CzcA | QAC21B_03355 | 59,599 | 150,270 |
|  | *czcD* | Metal cation efflux system protein CzcD | QAC21B_03356 |  |  |
|  | *czcO* | Putative oxidoreductase CzcO | QAC21B_03347 |  |  |
|  | *feoB* | Ferrous iron transport protein B homolog | QAC21B_03348 |  |  |
|  | *pcoA* | Copper resistance protein A | QAC21B_03343 |  |  |
|  | *insF* | Transposase InsF for insertion sequence IS3 | QAC21B_03328 |  |  |
|  | *vapB* | Virulence-associated protein B | QAC21B_03319 |  |  |
|  | *tnp1* | Transposase for insertion sequence-like element IS431mec | QAC21B_03318 |  |  |
|  | *tnpR* | Transposon Tn501 resolvase | QAC21B_03300 |  |  |
|  | *tnpA* | Transposase | QAC21B_02671 | 429,804 | 548,856 |
|  |  | Tellurite resistance protein | QAC21B_02636 |  |  |
|  |  | Putative transposase for transposon Tn903 | QAC21B_02464 | 717,902 | 724,817 |
|  | *mdtB* | Multidrug resistance protein MdtB | QAC21B_03217 | 2,449,583 | 2,459,341 |
|  | *mdtC* | Multidrug resistance protein MdtC | QAC21B_03216 |  |  |
|  | *insF* | Transposase InsF for insertion sequence IS3 | QAC21B_03130 | 2,555,928 | 2,561,196 |
|  | *tnpA1* | TnpA-A | QAC21B_03758 | 2,722,017 | 2,756,199 |
|  | *tnp2* | Transposase for insertion sequence-like element IS431mec | QAC21B_03765 |  |  |
|  | *int5* | Integrase | QAC21B_03774 |  |  |
|  | *vapB* | Virulence-associated protein B | QAC21B_03784 |  |  |
|  | *tnpR* | Transposon Tn501 resolvase | QAC21B_03812 | 2,788,199 | 2,797,179 |
|  | *insK* | Putative transposase InsK for insertion sequence element IS150 | QAC21B_03815 |  |  |
|  | *tnpA* | TnpA-A | QAC21B_00636 | 3,672,146 | 3,683,410 |
|  | *tnp1* | Transposase for insertion sequence-like element IS431mec | QAC21B_03607 | 3,952,839 | 3,992,385 |
|  | *tnpR* | Transposon Tn501 resolvase | QAC21B_03606 |  |  |
|  | *macB* | Macrolide export ATP-binding/permease protein MacB | QAC21B_03623 |  |  |
|  | IS*401* | Insertion element IS401 uncharacterized 12,4 kDa protein | QAC21B_03627 |  |  |
|  | *tnp1008* | Transposase | QAC21B_03901 |  |  |
|  | *tnp1* | Transposase for insertion sequence-like element IS431mec | QAC21B_03905 |  |  |
| *Acinetobacter bohemicus* ANC 3994^T^ |  | IS605 OrfB family transposase | F994_02674 | 2,075,536 | 2,122,615 |
| *Acinetobacter pakistanensis* ANC 5076 = KCTC 42081^T^ |  | MBL superfamily protein | SAMN05444586_1003173 | 299,813 | 315,463 |
|  |  | Acetyl esterase/lipase | SAMN05444586_100614 | 1,004,645 | 1,044,425 |
|  |  | Transposase IS6 family | SAMN05444586_104714 |  |  |
|  | *czcD* | Cobalt-zinc-cadmium efflux system protein | SAMN05444586_104712 |  |  |
|  | *czcA* | Cobalt-zinc-cadmium resistance protein CzcA | SAMN05444586_104711 |  |  |
|  | *feoA* | Ferrous iron transport protein A | SAMN05444586_10475 |  |  |
|  | *feoB* | Ferrous iron transport protein B | SAMN05444586_10474 |  |  |
|  | *czcO* | Predicted flavoprotein CzcO associated with the cation diffusion facilitator CzcD | SAMN05444586_10472 |  |  |
|  | *arsH* | Arsenical resistance protein ArsH | SAMN05444586_10471 |  |  |
|  |  | Predicted arabinose efflux permease, MFS family | SAMN05444586_10435 |  |  |
|  |  | Putative transposase | SAMN05444586_101865 | 1,263,313 | 1,271,902 |
|  | *tniB* | TniB protein | SAMN05444586_101866 |  |  |
|  | *tniQ* | TniQ protein | SAMN05444586_101868 |  |  |
|  |  | Putative transposase | SAMN05444586_10108 | 1,382,063 | 1,401,923 |
|  |  | Transposase IS6 family | SAMN05444586_102936 |  |  |
|  |  | OmpA family protein | SAMN05444586_10289 | 1,441,629 | 1,450,983 |
|  |  | Glutamin-(asparagin-)ase | SAMN05444586_101621 | 1,598,673 | 1,633,702 |
|  |  | Integrase | SAMN05444586_102069 | 2,993,905 | 3,010,214 |
|  | *cusS* | Two-component system, OmpR family, heavy metal sensor histidine kinase CusS | SAMN05444586_10528 | 3,428,660 | 3,507,668 |
|  | *cusR* | Two-component system, OmpR family, copper resistance phosphate regulon response regulator CusR | SAMN05444586_10529 |  |  |
|  | *copA* | Copper resistance protein A | SAMN05444586_105211 |  |  |
|  | *copB* | Copper resistance protein B | SAMN05444586_105212 |  |  |
|  |  | Transposase IS6 family | SAMN05444586_10982 |  |  |
|  |  | Transposase | SAMN05444586_10961 |  |  |
|  |  | Transposase | SAMN05444586_10515 |  |  |
|  |  | Transposase | SAMN05444586_103011 | 3,516,260 | 3,559,379 |
|  |  | Transposase | SAMN05444586_104110 | 3,577,878 | 3,745,643 |
|  |  | Transposase | SAMN05444586_104120 |  |  |
|  |  | Transposase | SAMN05444586_10912 |  |  |
|  |  | Transposase IS6 family | SAMN05444586_10815 |  |  |
|  |  | Transposase IS6 family | SAMN05444586_10891 |  |  |
|  |  | Integrase | SAMN05444586_10655 |  |  |
|  |  | Transposase | SAMN05444586_10656 |  |  |
|  |  | Transposase | SAMN05444586_10607 |  |  |
|  |  | Transposase | SAMN05444586_10734 | 3,809,907 | 3,826,362 |
|  |  | Transposase | SAMN05444586_10821 |  |  |
|  |  | Transposase IS66 family | SAMN05444586_10823 |  |  |
|  |  | Transposase IS630 family | SAMN05444586_11021 |  |  |
|  | *mdtC* | Multidrug efflux pump MdtC | SAMN05444586_100488 | 2,485,149 | 2,496,314 |
|  | *mdtB* | multidrug efflux pump MdtB | SAMN05444586_100489 |  |  |
|  |  | Transposase IS6 family | SAMN05444586_10541 | 2,586,374 | 2,592,167 |

**Supplementary figures**

**Figure S1** Phylogenetic placement of QAC-21b into a distinct cluster with strains of the species *A. bohemicus*. The phylogenetic tree was based on nearly full length nucleotide sequence of 16S rRNA gene. The evolutionary history was inferred by using the Maximum Likelihood method (Felsenstein 1981) based on the Kimura 2-parameter model (Kimura 1980) using a discrete gamma distribution to model evolutionary rate differences among sites [5 categories (+*G*)] and assuming some sites to be evolutionarily invariable ([+*I*]. The bootstrap values (≥ 70%) based on 100 re-samplings are shown above the branches. The analysis involved 87 nucleotide sequences. All positions containing gaps and missing data were eliminated. Scale, number of substitutions per site. Evolutionary analyses were conducted in MEGA7 (Kumar et al. 2016). Analysis was according to Pulami et al. (2021). Bar, 0.01 nucleotide substitutions per nucleotide position.

**Figure S2.** Phylogenetic placement of *A. bohemicus* QAC-21b into the species cluster of *A. bohemicus* within the genus *Acinetobacter*. Phylogenies calculated by Maximum Likelihood method (Felsenstein 1981) based on the General Time Reversible model (GTR; Nei and Kumar 2000) with a discrete Gamma distribution was used to model evolutionary rate differences among sites [5 categories (+G)] assuming some sites to be evolutionarily invariable (+I). Phylogenetic trees of (A) *rpoB* and (B) *gyrB* were based on total of 856 and 621 nucleotide positions in the final datasets. All positions containing gaps and missing data were eliminated. Bootstrap values (≥70%) after 100 resamplings are indicated at branch nodes; bar, number of substitutions per site. Evolutionary analyses were conducted in MEGA7 (Kumar et al. 2016). Circle represented nodes also present in phylogenetic tree calculated with the Neighbor-joining method (Saitou and Nei 1987).

**Figure S3** Phylogenetic tree based on partial amino acid sequence of *rpoB* showing the placement of QAC-21b into the species cluster of *A. bohemicus*. Phylogenetic analysis was inferred by using the Maximum Likelihood method based on the JTT matrix-based model (Jones et al. 1992) applying a discrete Gamma distribution to model evolutionary rate differences among sites (+G) assuming some sites to be evolutionarily invariable (+I). Bootstrap values (≥70%) based on 100 re-samplings are shown above the branches. The analysis involved total of 284 positions in the final dataset. Tree was calculated in MEGA7. Bar, rate of substitution per amino acid position.

**Figure S4** Phylogenetic tree based on partial amino acid sequence of *gyrB* showing the placement of QAC-21b into the species cluster of *A. bohemicus*. Phylogenetic analysis was inferred by using the Maximum Likelihood method based on the JTT matrix-based model, applying a discrete Gamma distribution to model evolutionary rate differences among sites (+G), assuming some sites to be evolutionarily invariable (+I). Bootstrap values (≥70%) based on 100 re-samplings are shown above the branches. The analysis involved a total of 205 positions in the final dataset. Tree was calculated in MEGA7. Bar, rate of substitution per amino acid position.

**Figure S5** (A) Heat map of average nucleotide identity (ANI) values of strain QAC-21b compared to *A. bohemicus* strains ANC 3994^T^ and KCTC 42081 and other related *Acinetobacter* strains. (B) Venn diagram depicting unique and shared genes between QAC-21b and *A. bohemicus* ANC 3994^T^ and KCTC 42081.

**Figure S6** Temperature dependent growth of QAC-21b analyzed by the spot assay method according to Pulami et al. (2021). 1, *A. baumannii* ATCC 19606^T^, 2, strain QAC-21b; 3, spotted 0.9% sodium chloride (NaCl) solution.

Analysis was performed after 24h of incubation

**Figure S7** Cupper tolerance of QAC-21b analyzed by the spot assay method according to Pulami et al. (2021). Copper tolerance was tested by pipetting 5 µL of serial dilution (10^0^–10^-3^ dilution, turbidity adjusted to standard 0.5 McFarland) of overnight cultured isolates in Muller Hinton agar plates (ROTH) supplemented with 4, 8, 12, 16, 20, 24, 32 and 36 mM CuSO_4_ 5H_2_O (adjusted to pH 7.2) (MERCK). All plates were incubated at 25°C in the dark and checked for growth after 24h of incubation. The lowest copper concentration that completely inhibited growth was considered as the MIC value. The growth was completely suppressed in concentration of 8 mM CuSO_4_ 5H_2_O and above (data not shown).

**Figure S8** Comparison of operon of class 1 integron (*intl1*) with QAACs efflux pump QacEdelta1 (*qacE∆1* gene) present in the chromosome of *Acinetobacter* strains. Figure and comparison were visualized using Easyfig v2.2.5 (Sullivan et al. 2011)

**
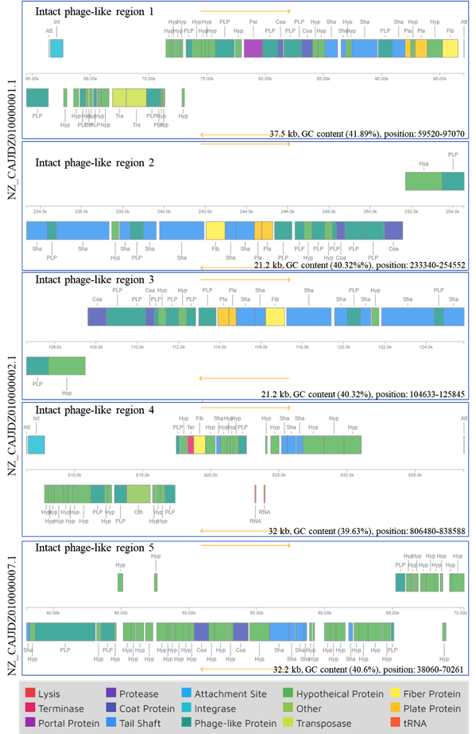
**

**Figure S9** Details of intact phage-like regions in *A. bohemicus* QAC-21b showing the characteristics of phage-related genes.


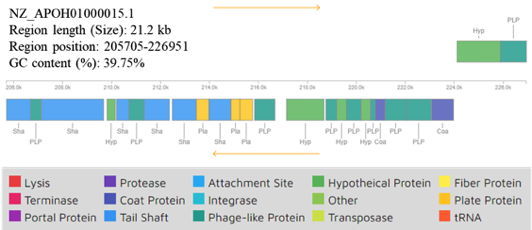


**Figure S10** Details of intact phage-like regions in *A. bohemicus* ANC 3994^T^ showing the characteristics of phage-related genes.

**Reference**

Abbas S, Ahmed I, Kudo T, et al (2014) Heavy metal-tolerant and psychrotolerant bacterium *Acinetobacter* pa*kistanensis* sp. nov. isolated from a textile dyeing wastewater treatment pond. Pakistan J Agric Sci 51:593–606

Arndt D, Grant JR, Marcu A, et al (2016) PHASTER: a better, faster version of the PHAST phage search tool. Nucleic Acids Res 44:W16–W21. https://doi.org/10.1093/nar/gkw387

Bertelli C, Laird MR, Williams KP, et al (2017) IslandViewer 4: expanded prediction of genomic islands for larger-scale datasets. Nucleic Acids Res 45:W30–W35. https://doi.org/10.1093/nar/gkx343

Bouvet PJM, Grimont PAD (1986) Taxonomy of the genus *Acinetobacter* with the recognition of *Acinetobacter* *baumannii* sp. nov., *Acinetobacter* *haemolyticus* sp. nov., *Acinetobacter* johnsonii sp. nov., and *Acinetobacter* *junii* sp. nov. and emended descriptions of *Acinetobacter* *calcoaceticus* and *Acinetobacter* *lwoffii*. Int J Syst Bacteriol 36:228–240. https://doi.org/10.1099/00207713-36-2-228

Choi JY, Ko G, Jheong W, et al (2013) *Acinetobacter* *kookii* sp. nov., isolated from soil. Int J Syst Evol Microbiol 63:4402–4406. https://doi.org/10.1099/ijs.0.047969-0

Felsenstein J (1981) Evolutionary trees from DNA sequences: a maximum likelihood approach. J Mol Evol 17:368–376. https://doi.org/10.1007/BF01734359

Jones DT, Taylor WR, Thornton JM (1992) The rapid generation of mutation data matrices from protein sequences. Bioinformatics 8:275–282. https://doi.org/10.1093/bioinformatics/8.3.275

Kimura M (1980) A simple method for estimating evolutionary rates of base substitutions through comparative studies of nucleotide sequences. J Mol Evol 16:111–120. https://doi.org/10.1007/BF01731581

Kumar S, Stecher G, Tamura K (2016) MEGA7: molecular evolutionary genetics analysis version 7.0 for bigger datasets. Mol Biol Evol 33:1870–1874. <https://doi.org/10.1093/molbev/msw054>

Nei M, Kumar S (2000) Molecular evolution and phylogenetics. Oxford university press

Nemec A, Radolfova-Krizova L (2016) *Acinetobacter* *pakistanensis* Abbas et al. 2014 is a later heterotypic synonym of *Acinetobacter* *bohemicus* Krizova et al. 2014. Int J Syst Evol Microbiol 66:5614–5617. https://doi.org/10.1099/ijsem.0.001530

Poirel L, Menuteau O, Agoli N, et al (2003) Outbreak of extended-spectrum β-lactamase VEB-1-producing isolates of *Acinetobacter* *baumannii* in a French hospital. J Clin Microbiol 41:3542–3547. https://doi.org/10.1128/JCM.41.8.3542-3547.2003

Pulami D, Schauss T, Eisenberg T, et al (2020) *Acinetobacter* *baumannii* in manure and anaerobic digestates of German biogas plants. FEMS Microbiol Ecol 96:fiaa176. https://doi.org/10.1093/femsec/fiaa176

Pulami D, Schauss T, Eisenberg T, et al (2021) Acinetobacter stercoris sp. nov. isolated from output source of a mesophilic German biogas plant with anaerobic operating conditions. Antonie van Leeuwenhoek, Int J Gen Mol Microbiol 114:235–251. <https://doi.org/10.1007/s10482-021-01517-7>

Thompson JD, Higgins DG, Gibson TJ (1994) CLUSTAL W: improving the sensitivity of progressive multiple sequence alignment through sequence weighting, position-specific gap penalties and weight matrix choice. Nucleic Acids Res 22:4673–4680. https://doi.org/10.1093/nar/22.22.4673

Zhou Y, Liang Y, Lynch KH, et al (2011) PHAST: A Fast Phage Search Tool. Nucleic Acids Res 39:W347-352. https://doi.org/10.1093/nar/gkr485
